# Changes in plant collection practices from the 16th to 21st centuries: implications for the use of herbarium specimens in global change research

**DOI:** 10.1093/aob/mcab016

**Published:** 2021-02-09

**Authors:** Mikhail V Kozlov, Irina V Sokolova, Vitali Zverev, Elena L Zvereva

**Affiliations:** 1 Department of Biology, University of Turku, Turku, Finland; 2 Herbarium, V. L. Komarov Botanical Institute, Professora Popova Str. 2, 197376, St. Petersburg, Russia

**Keywords:** Collection practices, global change, herbaria, herbivory, historical data, leaf size, reproduction, research biases, woody plants

## Abstract

**Background and Aims:**

Herbaria were recently advertised as reliable sources of information regarding historical changes in plant traits and biotic interactions. To justify the use of herbaria in global change research, we asked whether the characteristics of herbarium specimens have changed during the past centuries and whether these changes were due to shifts in plant collection practices.

**Methods:**

We measured nine characteristics from 515 herbarium specimens of common European trees and large shrubs collected from 1558 to 2016. We asked botanists to rank these specimens by their scientific quality, and asked artists to rank these specimens by their beauty.

**Key Results:**

Eight of 11 assessed characteristics of herbarium specimens changed significantly during the study period. The average number of leaves in plant specimens increased 3-fold, whereas the quality of specimen preparation decreased. Leaf size negatively correlated with leaf number in specimens in both among-species and within-species analyses. The proportion of herbarium sheets containing plant reproductive structures peaked in the 1850s. The scientific value of herbarium specimens increased until the 1700s, but then did not change, whereas their aesthetic value showed no systematic trends.

**Conclusions:**

Our findings strongly support the hypothesis that many characteristics of herbarium specimens have changed systematically and substantially from the 16th to 21st centuries due to changes in plant collection and preservation practices. These changes may both create patterns which could be erroneously attributed to environmental changes and obscure historical trends in plant traits. The utmost care ought to be taken to guard against the possibility of misinterpretation of data obtained from herbarium specimens. We recommend that directional changes in characters of herbarium specimens which occurred during the past 150‒200 years, primarily in specimen size and in the presence of reproductive structures, are accounted for when searching for the effects of past environmental changes on plant traits.

## INTRODUCTION

A growing number of studies are now using natural history collections to uncover changes in biota during the Anthropocene. Herbarium use, in particular, has diversified during the past century (Heberling *et al.*, 2019), and some scientists are calling for a more intensive utilization of these ‘windows into the past’ in global change research (Meineke and Davies, 2018; Meineke *et al*., 2018, 2019; Lang *et al.*, 2019). However, plants preserved in herbaria do not constitute a random sample from a population (Kozlov *et al.*, 2020); therefore, we cannot exclude the possibility that the temporal changes observed in the characteristics of herbarium specimens do not reflect solely the changes in biota. Rather, these changes may also mirror shifts in plant collection practices.

Botanists have discovered multiple biases in plant sampling for museum collections and in the subsequent accession and deaccession of these samples (Lavoie, 2013; Lang *et al.*, 2019, and references therein). For example, an analysis of approx. 5 million herbarium records identified spatial, temporal, trait, phylogenetic and collector biases (Daru *et al.*, 2018). Another study revealed strong collecting biases against introduced plants, plants with green or brown inflorescences and very small plants (Schmidt-Lebuhn *et al.*, 2013). All biases listed above affect the probability of collection of a certain plant species at a certain locality during a certain season. In addition, due to these biases, the characteristics of a herbarium specimen may not reflect the characteristics of the population from which this specimen had been collected. For example, some plants, such as the lianas of the genus *Mikania*, are represented in herbaria almost exclusively by their very tips, whereas lower leaves are rarely sampled even though they often differ markedly from upper leaves (Holmes, 1993). A recent study showed that insect herbivory measurements obtained from leaves of herbarium specimens are not proportional to the level of herbivory occurring in nature (Kozlov *et al.*, 2020). Some biases associated with the use of herbarium specimens can be moderated while searching for ecological patterns (Delisle *et al.*, 2003; Fithian *et al.*, 2015), but the number of studies accounting for these biases remains relatively low.

Intriguing and crucial questions regarding biases arise from the global change perspective. The most important of these is whether the difference between values of a certain trait (e.g. leaf size, specific leaf area, foliar nitrogen content or leaf area lost to insect herbivores) measured from herbarium specimens and values that occurred in natural populations at the time of plant collection (i.e. unavoidable sampling bias) remained constant for centuries or whether they changed with time. In particular, Meineke *et al.* (2018) mentioned that shifting collection practices could potentially give the appearance of changing herbivory through time, but no rigorous test for this hypothesis has been conducted.

The history of botanical research that is related to the collection and preservation of plants has involved many changes that could potentially affect the characteristics of herbarium specimens. These changes have been associated with both paradigm shifts (e.g. rejection of the typological concept when any specimen was thought to be an adequate representative of the species; Briggs, 2009) and with the development of plant preservation and storage techniques (Skvortsov, 1977; Egenberg and Moe, 1991; Willing and Willing, 1992). In particular, starting from the 18th century, botanists began to store pressed and mounted plants as loose-leaf pages rather than as bound volumes (Fleischer, 2017). Of equal importance, natural history collections evolved from curiosity cabinets, which served primarily aesthetic purposes (Ritterbush, 1969), meaning that old herbaria could have been created with high attention to their design. Some evidence (Irmscher, 1999) suggests that recent plant collectors have become less selective than they were in the 1800s, when the aesthetic appeal of a natural history specimen was presumably valued more highly than in recent times.

The ultimate goal of our study is to test the following hypotheses: (1) the characteristics of herbarium specimens that potentially reflect plant collection and preservation practices have changed substantially during the past centuries; and (2) the scientific value of herbarium specimens, as perceived by contemporary botanists, has increased during the past centuries, whereas the aesthetic value (beauty) of these specimens, as perceived by contemporary artists, has decreased, reflecting changes in the general approach to the creation of natural history collections. To verify these hypotheses, we analysed images of herbarium specimens collected from 1558 to 2016, focusing on those characters which could not be attributed to the impacts of environmental changes on plants. We paid particular attention to the changes that occurred during the past two centuries, because this period is usually considered by researchers who use herbarium specimens to address the global change issues (Willis *et al.*, 2017; Lang *et al.*, 2019; Meineke *et al.*, 2018). Based on our findings, we discuss how changes in collection practices may mimic the impacts of environmental changes on plants or may obscure historical patterns that actually existed in nature.

## MATERIALS AND METHODS

### Selection of study species

We searched for candidate plant species for this study among trees and large shrubs which had medium-sized leaves and which were widespread in northern Eurasia and sufficiently well represented in many herbaria. Based on the availability of images, we selected *Acer platanoides*, *Acer tataricum*, *Alnus glutinosa*, *Betula* spp. (*B. dahurica*, *B. pendula*, *B. platyphylla* and *B. pubescens*), *Corylus* spp. (*C. avellana*, *C. colchica*, *C. colurna*, *C. heterophylla* and *C. cornuta*), *Populus tremula*, *Pyrus* spp. (*P. communis*, *P. grossheimii* and *P. ussuriensis*), *Quercus robur*, *Sambucus nigra* and *Tilia platyphyllos*.

### Search for images of herbarium specimens

We intended to use images of 60 herbarium sheets of each of the ten species listed above, i.e. ten images per each of six pre-defined time periods (before 1775, 1775–1824, 1825–1874, 1875–1924, 1925–1974 and 1975–2018). We searched for the images of herbarium specimens from open access databases on the Global Plants on JSTOR (https://plants.jstor.org/), Virtual Herbaria JACQ (https://herbarium.univie.ac.at/database/) and on the websites of the large European herbaria and of one American herbarium. We used the species names as search terms and surveyed the images in the order in which they were listed by the search engine. We immediately rejected specimens which were either severely damaged during storage (i.e. their quality at the time of collecting was impossible to evaluate) or which did not have fully developed leaves (i.e. they had been collected in winter or early spring). We also excluded herbarium sheets which contained only seedlings or small juvenile plants. At this stage, we rejected approx. 5 % of the discovered images; subsequently, our selection was solely based on label data. Then we disregarded specimens with labels containing no information on the collection year. Among the dated specimens, we selected (on a first-found, first-used basis) one image by the combination of plant species × collector (or collection owner) × time period. At the next stage, some restrictions were relaxed and, in the absence of an alternative choice, we included in our list the second and, on occasion, even the third specimens from the same combination of plant species × herbarium × time period (or collector). At this stage, we also included the specimens with missing collection year for which we managed to identify the latest possible year based on the collector’s biography and other historical information (Stafleu and Cowan, 1976–1988). We also scanned several specimens stored at the Komarov Botanical Institute (St. Petersburg, Russia). Despite these efforts, we still failed to obtain sufficient numbers of images of the selected plant species collected before 1825; therefore, we used samples of smaller sizes (median number of images per plant species = 6) for the two oldest periods (Supplementary Data S1).

### Characteristics of the herbarium specimens

We quantified the following characteristics of each herbarium sheet: (1) paper size, measured as a diagonal of the sheet (only in 394 specimens scanned with a scale, mm); (2) number of objects (i.e. separate plant parts mounted on the same sheet); (3) number of leaves; (4) length of the lamina of the longest leaf (only in 394 specimens scanned with a scale, mm); (5) presence of reproductive structures (yes/no); (6) proportion of the herbarium sheet area covered by plant objects (visually estimated to the nearest 5 %); (7) proportion of overlapping leaves (0, 1–25, 26–50 and >50 %); (8) proportion of folded leaves (0, 1–25, 26–50 and >50 %); and (9) the level of leaf wrinkling (0, flat; 1, slightly wrinkled; 2, substantially wrinkled; 3, wrinkled and crumpled). All images were evaluated by the same person, who was not informed about the purpose of the study.

When the herbarium sheet contained two or more objects, we divided these objects into two groups: multiple plant parts intentionally mounted together or fragments of the same specimen (these had occasionally broken during processing and/or storage). Multiple plant parts that had apparently been intentionally combined were classified as either similar objects that jointly gave the same information on the plant species as each individual object (e.g. two branches without reproductive structures) or as different objects that jointly gave more information about the plant species than each individual object (e.g. vegetative branch, inflorescence and fruit). This classification was performed by I.V.S.

We have chosen woody plants for our study because their mature individuals are so large that the size of the herbarium specimens collected from trees and shrubs depends exclusively on the collector’s preferences. More generally, all studied characters, with the exception of leaf size, were unlikely to be affected by past environmental changes. Instead, they reflected the collector’s selection for a particular specimen and the quality of its preparation for press-drying.

### Editing of images of herbarium specimens

The images of all herbarium specimens were edited to avoid any subconscious impact of information about the collection date on the assessment of their scientific and aesthetic value. We removed all stamps, labels and barcodes from each herbarium sheet, added random numbers and changed the background colour to white. We also restored, whenever possible, the natural colours of leaves (Supplementary data Fig. S1). All images were edited using Adobe Photoshop CC. Using this software, we cut the original pictures to the same shape and size (650 × 1000 px), selected plant objects with the Lasso tool, placed them over a clean background, used the Eraser tool to remove the remaining parts of labels and finally adjusted colours of the background and of plant samples (using the Replace Colour function).

### Scientific value of herbarium specimens

The edited images of all herbarium sheets were evaluated for their scientific and aesthetic value. Twenty-three experienced botanists from 21 countries (see the Acknowledgements for the names of our respondents) ranked images of herbarium sheets (containing plants of the same species or species group) according to their subjective assessment of the scientific value of the photographed herbarium specimens. Each respondent was provided with an individual, fully randomized set of 60 images (i.e. six images of each of the ten plant species). Within each species, the highest rank (6) was assigned to the most valuable specimen and the lowest rank (1) to the least valuable specimen. We asked respondents to disregard damage that had occurred during plant storage in the herbarium and to assume that the six specimens of the same species had all been collected in one growing season from the same population. The latter information was, of course, not true. In fact, each of the six specimens in a group was collected during a different time period (see above), but the collection dates of these specimens were unknown to the respondents. This blinding was applied to prevent biased evaluation of the images.

### Aesthetic value of herbarium specimens

Twenty-one artists from eight countries (see the Acknowledgements for the names of those respondents who did not wish to remain anonymous) ranked the same images (see above) according to their subjective assessment of the aesthetic value (beauty) of the herbarium specimens. Many, but not all, of these artists work on botanical art.

### Data analysis

For the first analysis, we averaged the values of each studied character across all herbarium specimens collected during each 10-year-long period, from 1550–1559 to 2010–2016. We selected this approach to minimize the effects of uneven distribution of herbarium specimens among the collection years, especially between 1550 and 1825. We disregarded three decades for which we obtained only one or two herbarium sheets, and we regressed the remaining means (which all fitted a normal distribution) by the median years of these periods. We compared linear and quadratic regression models for each response variable using an online calculator (graphpad.com/quickcalcs/aic2/), and we selected the best-fit model based on the Akaike information criterion (Motulsky and Christopoulos, 2003).

The second analysis was based on unaggregated data on specimens collected from 1826 to 2016, i.e. within the period which is usually considered in the global change research. We used a mixed model analysis of covariance (ANCOVA; SAS GLIMMIX procedure, type III tests; SAS Institute, 2009) with different model statements: normal distribution with identity link function for sheet area, area covered by plant specimen, number of leaves, and scientific and aesthetic values of herbarium specimens; Poisson distribution with log link function for the number of objects; binomial distribution with logit link function for the presence of generative structures; and multinomial distribution with cumlogit link function for the proportions of folded and overlapped leaves and for the level of leaf wrinkling (SAS Institute, 2009). In all these analyses, we considered plant species and the source herbarium as random effects, and collection year as a covariate. The unbalanced sampling design did not allow for testing a hypothesis that temporal trends in characteristics of plant specimens differ among herbaria. To facilitate accurate *F*-tests of the effect of study year, we adjusted the standard errors and denominator degrees of freedom in all our analyses by the latest version of the method described by Kenward and Roger (2009). The significance of the random factor was evaluated by calculating the likelihood ratio and testing it against the χ ^2^ distribution (as described in Littell *et al.*, 2006).

The relationships between the ranks of scientific and aesthetic value of herbarium specimens were quantified by Spearman rank correlation coefficients. The correlation coefficients were compared using an online calculator (vassarstats.net/rdiff.html). We explored the relationships between leaf size and leaf number at two levels. The species-level analysis was based on the values of both characters averaged by plant species, whereas the specimen-level analysis was based on the values standardized (to mean = 0 and s.d. = 1) by plant species to remove the among-species differences in leaf size and number.

## RESULTS

### Overview of the data

We analysed the images of the 515 herbarium sheets, which were attributed to >374 collectors and/or collection owners, from 19 herbaria worldwide (Supplementary Data S1). The largest numbers of images originated from the Muséum National d’Histoire Naturelle, Paris (117), Moscow State University (82) and the Nationaal Herbarium Nederland, Leiden University branch (63), because these collections are completely or largely digitalized. The size of a herbarium sheet in our sample varied from 155 × 195 mm to 325 × 503 mm. The selected herbarium sheets included from one to 12 separate objects (median value = 1) and from one to 117 leaves (median value = 16). Among the 270 herbarium sheets with multiple objects, 132 sheets contained different objects, 104 sheets contained similar objects and the remaining 34 sheets contained fragments of the same specimen. Plant specimen(s) covered from 10 to 80 % of a sheet (median value = 40 %); over two-thirds (68.6 %) of the sheets contained plant reproductive structures.

### Relationships between size and number of leaves in a herbarium specimen

**Fig. 1.**
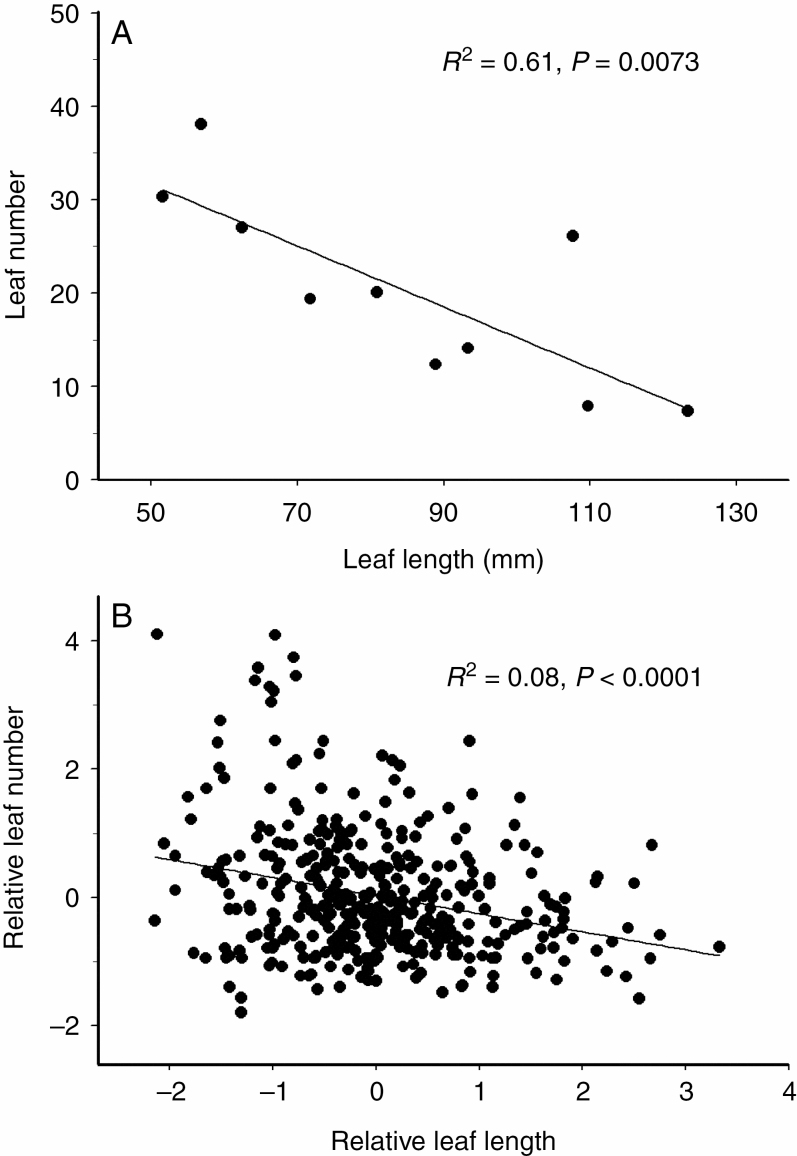
Correlation between leaf size (measured as the length of the lamina of the largest leaf) and the number of leaves in a herbarium specimen: (A) at the level of plant species (absolute values); and (B) at the level of plant individuals (values standardized by plant species).

Within the specimens included in the analysis, the number of leaves is generally smaller for large-leaved tree species than for small-leaved species (Fig. 1A). Similarly, within all study species, the correlation between leaf size and number was negative, although it reached the conventional level of statistical significance in only four of the ten species/species groups: *A. tataricum*, *Betula* spp., *P. tremula* and *T. platyphyllos* (Table 1). The standardized values of leaf size were negatively correlated with the standardized values of leaf number across a combined sample of all study species (Fig. 1B).

**Table 1.** Within-species correlation between the length of the lamina of the largest leaf and the number of leaves in a herbarium sheet

Plant species	*r*	*n*	*P*
*Acer platanoides*	–0.16	37	0.34
*Acer tataricum*	–0.34	37	0.04
*Alnus glutinosa*	–0.31	37	0.06
*Betula* spp.	–0.68	45	<0.0001
*Corylus* spp.	–0.20	34	0.27
*Populus tremula*	–0.34	41	0.03
*Pyrus* spp.	–0.22	37	0.19
*Quercus robur*	–0.05	46	0.72
*Sambucus nigra*	–0.12	37	0.49
*Tilia platyphyllos*	–0.35	43	0.02

*r*, Pearson product–moment correlation coefficient; *n*, sample size; *P*, probability level.

### Temporal changes in the characteristics of the herbarium specimens

**Fig. 2.**
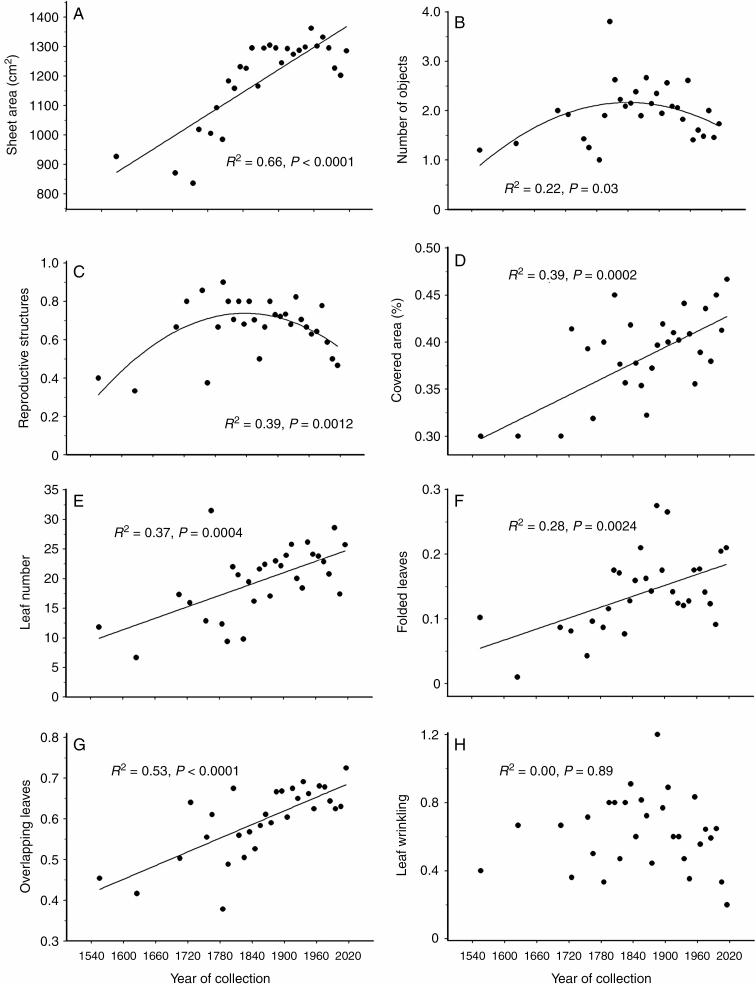
Temporal changes in the characteristics of herbarium specimens (averaged by decades from three to 31 herbarium sheets; for exact values, consult Supplementary Data S1). (A) The area of a herbarium sheet; (B) the number of objects mounted on a herbarium sheet; (C) the proportion of herbarium sheets containing plant reproductive structures; (D) the proportion of a herbarium sheet area covered by plant parts; (E) the number of leaves; (F) the proportion of folded leaves; (G) the proportion of overlapping leaves; and (H) an arbitrary ranking of the level of leaf wrinkling.

Seven of the nine assessed characteristics of herbarium specimens changed significantly from 1550 to 2016. The average area of a herbarium sheet increased by 62% (Fig. 2A). Both the number of objects mounted on a herbarium sheet (Fig. 2B) and the proportion of herbarium sheets containing reproductive structures (Fig. 2C) showed dome-shaped temporal patterns, attaining the highest values in the middle of the 19th century. The proportion of a herbarium sheet covered by plant specimens (Fig. 2D) and the number of leaves (Fig. 2E), as well as the proportions of folded (Fig. 2F) and overlapping (Fig. 2G) leaves, increased with the collection year. The relative (per unit of herbarium sheet area) numbers of objects and plant leaves showed the same patterns as their absolute values, although the significance of the effects was smaller (*R*^2^ = 0.13, *P* = 0.056 and *R*^2^ = 0.20, *P* = 0.055, respectively) when compared with the effects observed in absolute values (Fig. 2B, E). The proportion of herbarium sheets with different objects peaked around 1810s (*R*^2^ = 0.31, *P* = 0.0066), whereas the proportion of herbarium sheets with similar objects tended to increase with the collection year (*R*^2^ = 0.11, *P* = 0.07). Leaf size (*R*^2^ = 0.05, *P* = 0.26) and wrinkling (Fig. 1H) showed no temporal changes. However, when leaf number was included as an explanatory variable, the regression analysis revealed an increase in leaf size with the increase in collection year (*R*^2^ = 0.26, *P* = 0.0002).

The additional analysis of specimens collected from 1826 to 2016 (Table 2) generally confirmed the patterns outlined above (Fig. 2). The two exceptions were the area of a herbarium sheet and the proportion of herbarium sheets containing reproductive structures, which did not change systematically during the past 200 years. However, the proportion of herbarium sheets containing reproductive structures significantly decreased during the past 140 years, i.e. between 1876 and 2016 (*F*_1, 297_ = 3.73, *P* = 0.05).

**Table 2.** Sources of variation in characteristics of herbarium specimens collected from 1826 to 2016 (SAS GLIMMIX procedure, type III sum of squares).

Character	Year	Plant species	Herbarium
Sheet area	*F*_1, 315.5_ = 2.34, *P* = 0.13	χ ^2^ = 0.00, *P* = 0.99	χ ^2^ = 202.8, *P* < 0.0001
Number of objects	*F*_1, 398_ = 13.0, *P* = 0.0004	χ ^2^ = 4.81, *P* = 0.01	χ ^2^ = 0.01, *P* = 0.46
Generative structures	*F*_1, 398_ = 2.37, *P* = 0.12	χ ^2^ = 31.8, *P* < 0.0001	χ ^2^ = 12.3, *P* = 0.0002
Covered area	*F*_1, 329_ = 8.95, *P* = 0.0030	χ ^2^ = 9.57, *P* = 0.0010	χ ^2^ = 1.59, *P* = 0.10
Leaf number	*F*_1, 284.1_ = 6.17, *P* = 0.01	χ ^2^ = 170.7, *P* < 0.0001	χ ^2^ = 0.16, *P* = 0.34
Folded leaves	*F*_1, 245.2_ = 0.10, *P* = 0.75	χ ^2^ = 20.6, *P* < 0.0001	*χ* ^2^ = 0.00, *P* = 0.49
Overlapped leaves	*F*_1, 396_ = 13.2, *P* = 0.0003	χ ^2^ = 19.9, *P* < 0.0001	χ ^2^ = 7.13, *P* = 0.0038
Leaf wrinkling	*F*_1, 396_ = 4.51, *P* = 0.03	χ ^2^ = 47.4, *P* < 0.0001	χ ^2^ = 13.1, *P* = 0.0001
Scientific value	*F*_1, 392.9_ = 1.59, *P* = 0.21	χ ^2^ = 0.00, *P* = 0.99	χ ^2^ = 13.0, *P* = 0.0003
Aesthetic value	*F*_1, 355.8_ = 2.32, *P* = 0.13	χ ^2^ = 0.00, *P* = 0.99	χ ^2^ = 1.98, *P* = 0.16

### Temporal changes in the scientific and aesthetic value of the herbarium specimens

**Fig. 3.**
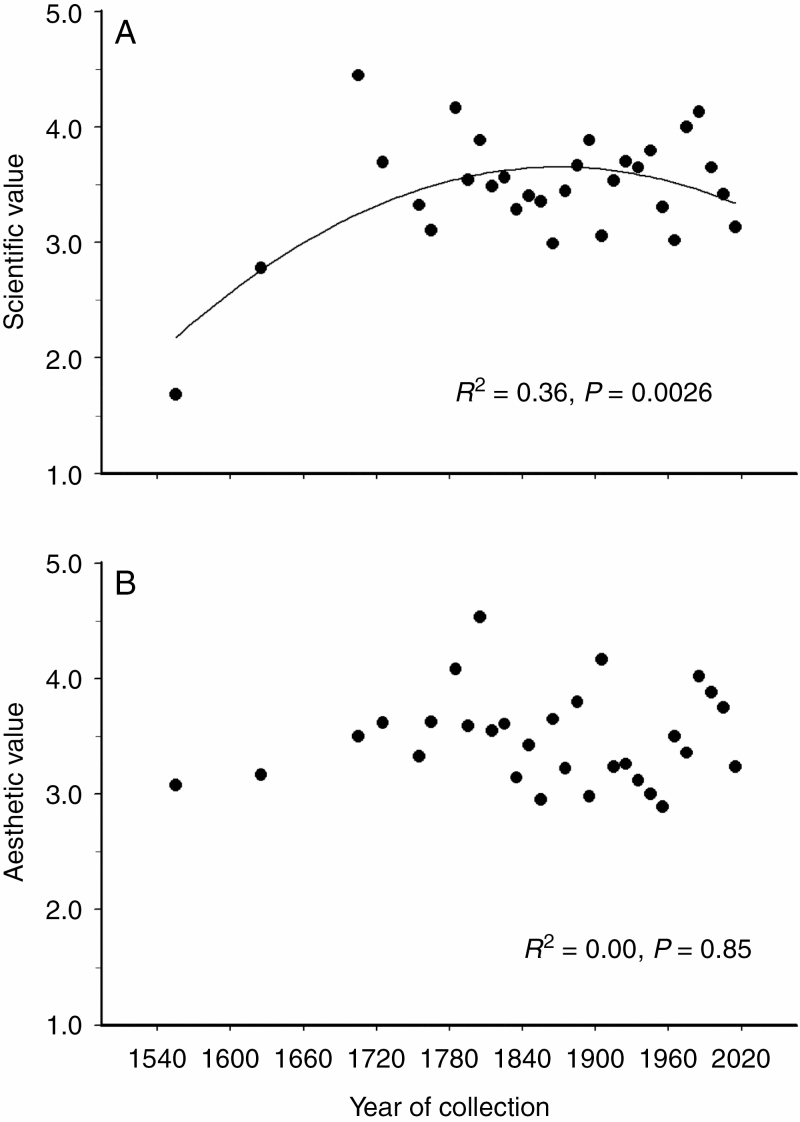
Temporal changes in characteristics of herbarium specimens (averaged by decades from three to 31 herbarium sheets). (A) An arbitrary ranking of the scientific value; and (B) an arbitrary ranking of the aesthetic value.

The scientific value of the herbarium specimens showed a dome-shaped temporal pattern (Fig. 3A). However, after exclusion of the ‘En Tibi’ herbarium (dated 1558), the scientific value showed no systematic temporal changes (*R*^2^ = 0.00, *n* = 29, *P* = 0.84). The scientific value of the same 120 images was ranked similarly by different scientists (*r*_S_ = 0.61, *n* = 180, *P* < 0.0001).

The aesthetic value of herbarium specimens did not change with time (Fig. 3B) and weakly but positively correlated with the scientific value of the same specimens (*r*_S_ = 0.15, *n* = 515, *P* = 0.0006). The aesthetic value of the 120 images was ranked similarly by different artists (*r*_S_ = 0.31, *n* = 120, *P* < 0.0001); however, the discrepancies were significantly larger for the evaluation of an image between two artists than between two scientists (*Z* = 3.65, *P* = 0.0003).

### Variation among plant species and among herbaria

Six of ten characters of herbarium specimens varied among plant species, and five of ten characters varied among herbaria (Table 2). Within ten herbaria, from which we analysed >10 herbarium specimens, the average area of a herbarium sheet varied from 1066 cm^2^ in the Royal Botanical Gardens Kew (UK) to 1581 cm^2^ in the Naturalis (the Netherlands). The percentage of herbarium sheets containing reproductive structures was lowest (38 %) in the Komarov Botanical Institute (Russia) and highest (86 %) in the Naturalis. The proportion of overlapping leaves varied from 55 % in the Royal Botanical Gardens Kew to 75 % in the Komarov Botanical Institute. The leaves were flat (i.e. not wrinkled) in 67 % of specimens from the New York Botanical Garden (USA) but only in 26 % of specimens from the Komarov Botanic Institute. The scientific value was lowest (average rank 2.88 on a scale from 1 to 6) in specimens from the Komarov Botanical Institute and highest (average rank 4.27) in specimens from the New York Botanical Garden.

## DISCUSSION

### Changes in collection practices reflected in herbarium specimens

Our findings strongly support the hypothesis that many characteristics of plant specimens preserved in herbaria have changed systematically and substantially during almost five centuries of the existence of scientific collections of press-dried plants. For example, the average number of plant leaves per herbarium sheet increased 3-fold, from eight in the 1550s to 24 in the 2010s, whereas the area of a herbarium sheet increased by only 1.6-fold. The increase in herbarium sheet area from the 16th to 18th century may be partly explained by the decreasing cost of paper over the centuries, but increases in the number of leaves and in the relative area of a herbarium sheet covered by plant objects are likely to be associated with the growing appreciation of the importance of within-plant variations in leaf size and shape, as well as of the traits of stems (including branching pattern), for the adequate morphological description of a species.

The increase in the size of a plant specimen, as reflected by the increases in both the number of leaves and the area covered by it, made specimen preparation for press-drying a more demanding task. The overall impression is that, although the folded leaves decreased both the scientific and the aesthetic value of the herbarium specimens, many botanists were not motivated to invest more time in spreading the leaves, as indicated by the increase in the proportion of folded leaves that paralleled the increase in leaf numbers. At the same time, the level of leaf wrinkling showed no temporal trend, indicating that the strength of pressing applied to the collected specimens at the time of their drying has not changed over the centuries.

The number of objects mounted on a herbarium sheet and the proportion of sheets containing reproductive structures both peaked around the 1850s. We suggest that the mounting of different objects, which were probably collected from different plant individuals and/or at different dates (to include leaves, flowers and fruits on the same sheet), may reflect an attempt to increase the scientific value of herbaria, a practice that attained its maximum in the early 19th century. In contrast, mounting of similar plant parts (usually branches) on the same herbarium sheet is increasingly common among contemporary botanists, presumably because processing of several small (thin) branches is simpler relative to one large (thick) branch and saves valuable storage space.

The earlier herbaria were often perceived as art objects. For example, the ‘En Tibi’ herbarium is a Renaissance masterpiece of art and science (Stefanaki *et al.*, 2019). In several old herbaria, cartouches for plant names and/or ornaments were added to each sheet, and some of them created the impression that the plant specimens they carried were growing from a vase (Fleischer, 2017). Linnaeus cut these sheets down to a size that would fit into his cabinet, indicating that aesthetic reasons were of little importance to him (Müller-Wille, 2006).

Nevertheless, the aesthetic reasons still were taken into account by many botanists, as indicated by a significant, albeit weak, positive correlation between the scientific value and the aesthetic value of herbarium specimens. However, contrary to expectations, we did not discover any temporal shift in the aesthetic value of herbarium specimens. This finding suggests that, as in the case of selectivity with respect to insect damage (Kozlov *et al.*, 2020), the importance of aesthetic reasons varied among collectors, and this variation appeared much greater than the temporal shift in the importance of aesthetic reasons in the selection and preparation of herbarium specimens. The importance of aesthetic reasons can also depend on external circumstances and would be lower in specimens collected during short-term excursions to remote localities than in specimens sampled next to a botanist’s home. Last, but not least, the lower repeatability of assessments made by artists, as compared with botanists, suggests that the estimates of the aesthetic value are more subjective than are the estimates of the scientific value of herbarium specimens.

### Implications for global change research

We found that the proportion of herbarium sheets containing plant reproductive structures increased until the middle of the 19th century and then decreased again. The increasing branch of this dome-shaped curve probably reflects the growing appreciation of the importance of reproductive structures for plant taxonomy during the 18th to 19th centuries, whereas the reasons for the significant decrease in the proportion of herbarium specimens bearing reproductive structures during the past 150 years remain obscure. This pattern may reflect our selection of plant species among common trees, as these can be reliably identified without analysis of their reproductive structures. Still, these changes may have a substantial impact on the conclusions derived from studies of herbarium specimens.

Reproduction is a resource-demanding process which bears some costs, i.e. it competes with other plant functions. A wealth of studies has demonstrated that the production of fruits is correlated with reduced vegetative growth due to internal reallocation of limited resources (Obeso, 2002, and references therein). The result is that many traits, including leaf size, shoot length, number of buds and length of internodes, often differ between the vegetative and generative shoots of the same plant individual (Tuomi *et al.*, 1982; Obeso, 1997; Kozlov and Zvereva, 2004). Therefore, studies addressing historical changes in plant morphology are likely to detect an increase in leaf size and shoot length in woody plants (or at least in trees and shrubs included in our study) due to a decrease in the proportion of plant specimens with generative shoots during the past 150 years. To prevent false discoveries, the vegetative and generative shoots of herbarium specimens should be measured and analysed separately.

The decrease in the proportion of herbarium sheets containing reproductive structures may also indicate that, in the 20th–21st centuries, the botanists collected juvenile trees more frequently than they did in the 19th century. Woody species commonly exhibit changes in morphological, physiological and biochemical characteristics of their leaves with increased size or age of a plant (reviewed by Hackett, 1985; Boege and Marquis, 2005; Barton and Koricheva, 2010), and juvenile individuals often suffer greater foliar damage by herbivores than is seen in mature (reproducing) plants (Fox and Morrow, 1983; Niesenbaum, 1992; Stone and Bacon, 1994). As a result, an increase in the proportion of juvenile plants among herbarium specimens could create false patterns of changes in leaf characteristics and in herbivory, which could be erroneously interpreted as consequences of climate warming. Unfortunately, a branch lacking flowers/seeds may have been collected from both juvenile and mature tree individuals. Therefore, we recommend that the characteristics of tree leaves that depend on plant age, including herbivory, be measured only from herbarium specimens bearing reproductive structures.

The increase in the size of tree branches preserved in herbaria during the past centuries, as reflected by an increase in leaf number and in the area covered by plants, may also affect the temporal patterns revealed in several plant traits. In particular, the imperative to collect a branch with a large number of leaves may lead to a preference for branches with smaller than average leaves. This prediction was confirmed by the negative correlation between leaf size and leaf number in our sample of herbarium specimens at both the species and the specimen levels. The selection of branches with smaller than average sizes of leaves in large-leaved plants explains e.g. why the leaf lengths of *Tilia* spp. measured from herbarium specimens were typically smaller than those based on measurements of both herbarium specimens and living plants (Corney *et al.*, 2012). We assume that the selection for branches with small leaves could be especially strong in large-leaved plants, whose leaves approach the size of a herbarium sheet. Most importantly, accounting for the number of plant leaves in a herbarium specimen revealed an overall increase in the leaf size of our study trees during the past centuries, which was not detected when the year of collection was used as the only explanatory variable in the regression analysis.

The substantial increase in the number of leaves in herbarium specimens between 1558 and 2016 may also have affected the levels of insect herbivory measured from these specimens. Botanists have always been advised to collect specimens that bear no or few signs of damage (Greville, 1840; Bailey, 1899; Pearsall, 2015), and an earlier study demonstrated that both collectors and curators generally preferred specimens with less leaf damage (Kozlov *et al.*, 2020). Obviously, finding a large (50‒100 leaves) branch with no or minor leaf damage is more challenging than selecting a small (5‒15 leaves) branch with no traces of insect feeding. In combination with the presumably lower level of care taken recently by botanists to obtain perfect herbarium specimens, as suggested by the 4-fold increase in the proportion of folded leaves during the past centuries, the data collected from older (smaller) herbarium specimens are likely to underestimate the foliar damage by insects to a greater extent when compared with data collected from currently sampled (larger) specimens. Thus, the observed changes in plant collection practices may mimic the expected (e.g. Ayres and Lombardero, 2000; O’Connor *et al.*, 2009; DeLucia *et al.*, 2012) contemporary increase in herbivory. This effect may be alleviated by using the number of leaves in a herbarium specimen as a covariate when analysing leaf sizes and herbivory levels, although we are uncertain that this approach could fully compensate for presumed temporal shifts in the selectivity practiced by plant collectors.

### Conclusion

We revealed substantial changes in multiple characteristics of herbarium specimens of common European leaf-bearing trees and large shrubs collected from 1558 to 2016. With the exception of leaf size, these changes could be explained by changes in plant collection and preservation practices alone, rather than by environmental changes. This discovery has direct implications for global change research, because the historical patterns in both plant traits and levels of herbivory, derived from studies of herbarium specimens, may reflect shifts in plant collection and preservation practices rather than the effects of past environmental changes on plant characteristics. Similarly, the changes in plant collection practices may prevent the identification of actual temporal trends in plant traits. Therefore, the utmost care ought to be taken to guard against the possibility of misinterpretation of morphological, ecological and environmental data obtained from historical herbarium specimens. We recommend that directional changes in characters of herbarium specimens which occurred during the past 150‒200 years, primarily in specimen size and in the presence of reproductive structures, are accounted for when searching for the effects of past environmental changes on plant traits.

## SUPPLEMENTARY DATA

Supplementary data are available online at https://academic.oup.com/aob and consist of the following. Figure S1: examples of non-edited and edited images of herbarium specimens. Data S1: characteristics of herbarium specimens used in the study.

mcab016_suppl_Supplementary_S01
