# Supplementary material for: Changes in plant collection practices from the 16th to 21st centuries: implications for the use of herbarium specimens in global change research
Source: Ann Bot. 2021 Feb 9;127(7):865–73. doi: 10.1093/aob/mcab016 (PMC8225282; doi:10.1093/aob/mcab016)
Supplement: mcab016_suppl_Supplementary_S01 [file mcab016_suppl_Supplementary_S01.doc]

**Changes in plant collection practices from the 16^th^ to 21^st^ centuries: implications for the use of herbarium specimens in global change research**

Mikhail V. Kozlov^1*^, Irina V. Sokolova^2^, Vitali Zverev^1^, and Elena L. Zvereva^1^

^1^ Department of Biology, University of Turku, 20014 Turku, Finland

^2^ Herbarium, V. L. Komarov Botanical Institute, Professora Popova Str. 2, 197376 St. Petersburg, Russia

* Correspondence author. E-mail: mikoz@utu.fi. ORCID: 0000-0002-9500-4244.

**Supplementary material**


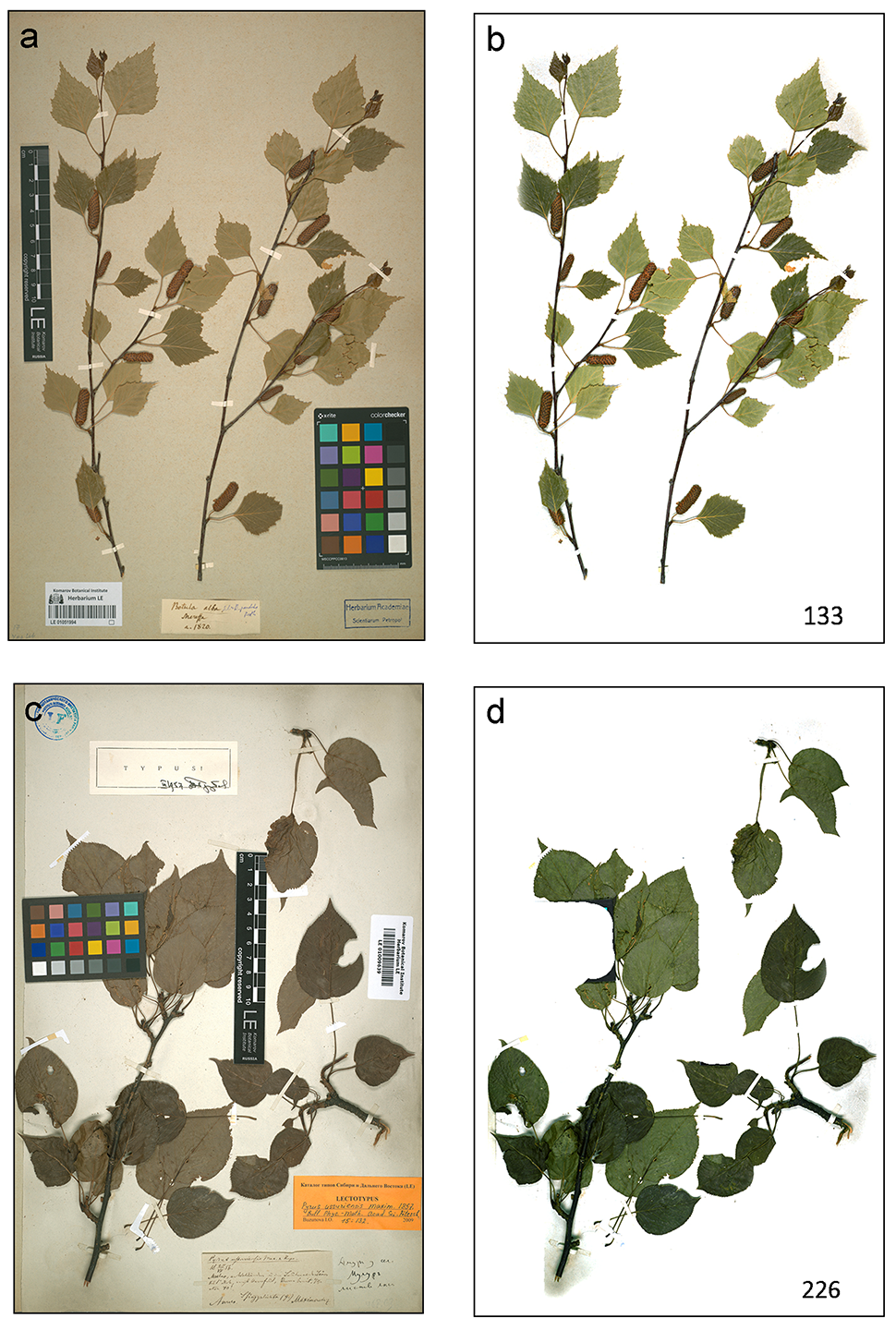


**Fig. S1**. Examples of non-edited (a, c) and edited (b, d) images of herbarium specimens.

**Data S1**. Characteristics of herbarium specimens used in the study.

Column 1: random number, which was used as the sole identifier of the image at all stages of data collection.

Column 2: species code (acepla, *Acer platanoides*; acetat, *A. tataricum*; alnglu, *Alnus glutinosa*; betula, *Betula* *pendula* & *B. pubescens*; corylu, *Corylus* *avellana*, *C. colchica*, *C. colurna*, *C. heterophylla*, & *C. cornuta*; poptre, *Populus tremula*; pyrus, *Pyrus* *communis*, *P. grossheimii*, & *P. ussuriensis*; querob, *Quercus robur*; sambuc, *Sambucus nigra*; tilpla, *Tilia platyphyllos*.

Column 3: collector or collection owner, if collector is unknown.

Column 4: collecting year, as identified from herbarium specimen and/or from other data sources (b., before; c., circa).

Column 5: collecting year, as used in data analysis.

Column 6: depository; acronyms follow Thiers, B. (2020) *Index Herbariorum: A global directory of public herbaria and associated staff*. *New York Botanical Garden's Virtual Herbarium.* http://sweetgum.nybg.org/science/ih/.

Column 7: specimen identifier (barcode or collection number).

Column 8: size of herbarium sheet (diagonal, mm).

Column 9: average scientific value of the specimen (1, the lowest; 6, the highest).

Column 10: average aesthetic value of the specimen (1, the lowest; 6, the highest).

Column 11: number of objects (i.e. of separate plant parts) mounted on a herbarium sheet.

Column 12: types of objects (0, similar objects intentionally mounted together that jointly gave the same information on the plant species as each individual object, e.g. two branches without reproductive structures; 1, different objects intentionally mounted together that jointly gave more information about the plant species than each individual object, e.g. vegetative branch, inflorescence, and fruit; 2, the specimen had occasionally broken during processing and/or storage).

Column 13: number of leaves (in all objects mounted on a herbarium sheet).

Column 14: length of lamina of the largest leaf (mm).

Column 15: presence of reproductive structures (0, no; 1, yes) on a herbarium sheet.

Column 16: proportion of herbarium sheet area covered by plant objects (%).

Column 17: proportion of overlapping leaves (0, no overlapping leaves; 1, 1–25%; 2, 26–50%; 3, >50% of leaves overlap each other).

Column 18: proportion of folded leaves (0, no folded leaves; 1, 1–25%; 2, 26–50%; 3, >50% of leaves are folded).

Column 19: average leaf wrinkling (0: flat, 1: slightly wrinkled, 2: substantially wrinkled, 3: wrinkled and crumpled).

| **1** | **2** | **3** | **4** | **5** | **6** | **7** | **8** | **9** | **10** | **11** | **12** | **13** | **14** | **15** | **16** | **17** | **18** | **19** |
| --- | --- | --- | --- | --- | --- | --- | --- | --- | --- | --- | --- | --- | --- | --- | --- | --- | --- | --- |
| 1 | corylu | Bauhin | b.1624 | 1624 | BAS | . | 433.2 | 6.00 | 4.00 | 2 | 1 | 12 | 91.2 | 1 | 40 | 3 | 0 | 1 |
| 2 | sambuc | Borzov | 1927 | 1927 | MW | MW0522490 | 526.5 | 4.00 | 1.50 | 3 | 0 | 10 | 119.1 | 1 | 60 | 3 | 1 | 0 |
| 3 | betula | Desplantes | 1934 | 1934 | P | P00528847 | . | 3.50 | 3.50 | 2 | 1 | 27 | . | 1 | 20 | 3 | 1 | 0 |
| 4 | acepla | Sherard | b.1728 | 1728 | OXF | . | 428.3 | 2.00 | 4.00 | 2 | 0 | 5 | 124.9 | 0 | 50 | 3 | 1 | 1 |
| 5 | sambuc | Vaillant | b.1722 | 1722 | P | P03707196 | . | 2.75 | 2.75 | 1 | . | 1 | . | 1 | 20 | 0 | 1 | 0 |
| 6 | pyrus_ | Meyer | 1913 | 1913 | P | P03132067 | . | 4.00 | 1.00 | 1 | . | 45 | . | 0 | 40 | 3 | 1 | 0 |
| 7 | corylu | Bartholomew et al. | 1984 | 1984 | L | L.3738416 | 581.1 | 3.00 | 3.00 | 2 | 2 | 14 | 128.3 | 1 | 50 | 3 | 3 | 0 |
| 8 | acetat | . | 2001 | 2001 | P | P04795623 | . | 3.50 | 3.50 | 1 | . | 8 | . | 1 | 30 | 3 | 2 | 1 |
| 9 | pyrus_ | Behrendsen | 1873 | 1873 | U | U.1552116 | 569.7 | 4.50 | 3.50 | 1 | . | 5 | 46.5 | 1 | 15 | 3 | 0 | 0 |
| 10 | tilpla | Reshetnikova | 2001 | 2001 | MW | MW0435836 | 502.5 | 6.00 | 5.00 | 1 | . | 8 | 118.8 | 1 | 55 | 2 | 0 | 0 |
| 11 | pyrus_ | Danty d’Isnard | b.1743 | 1743 | P | P03141081 | . | 5.00 | 3.57 | 1 | . | 83 | . | 1 | 50 | 3 | 1 | 2 |
| 12 | betula | Candolle | b.1822 | 1822 | P | P00528843 | . | 5.50 | 2.50 | 2 | 1 | 12 | . | 1 | 25 | 3 | 1 | 0 |
| 13 | tilpla | Schleicher | b.1824 | 1824 | GDC | G00209197 | 463.9 | 4.60 | 3.40 | 1 | . | 7 | 109.7 | 1 | 55 | 3 | 0 | 1 |
| 14 | betula | Mérat | 1819 | 1819 | P | P00528849 | 508.9 | 6.00 | 4.50 | 1 | . | 38 | 55.5 | 1 | 40 | 3 | 1 | 0 |
| 15 | pyrus_ | David | 1865–1874 | 1874 | P | P03132069 | 597.8 | 3.00 | 2.00 | 2 | 2 | 22 | 49.7 | 0 | 40 | 3 | 1 | 2 |
| 16 | acetat | van Hattum | 1946 | 1946 | L | L.3702255 | 576.9 | 3.50 | 2.50 | 2 | 2 | 12 | 83.1 | 1 | 35 | 3 | 0 | 1 |
| 17 | querob | Scheppig | 1859 | 1859 | AMD | AMD.74082 | . | 4.50 | 4.00 | 1 | . | 12 | . | 0 | 40 | 3 | 2 | 0 |
| 18 | acetat | Domin & Krajina | 1929 | 1929 | MW | MW0782942 | 529.9 | 5.00 | 3.00 | 2 | 0 | 30 | 69.3 | 1 | 40 | 3 | 1 | 1 |
| 19 | corylu | Baranova & Pudnova | 1982 | 1982 | MW | MW0049870 | 520.8 | 1.50 | 3.00 | 2 | 0 | 33 | 94.5 | 1 | 50 | 3 | 1 | 1 |
| 20 | querob | Sokolova | 1936 | 1936 | MW | MW0315019 | 405.7 | 2.50 | 2.50 | 2 | 0 | 31 | 78.5 | 1 | 50 | 3 | 1 | 1 |
| 21 | alnglu | Matthews | 1945 | 1945 | NY | 02471632 | 515.0 | 3.50 | 4.50 | 2 | 0 | 18 | 65.6 | 1 | 40 | 2 | 1 | 0 |
| 22 | acetat | Merello et al. | 1999 | 1999 | E | E00281561 | 494.5 | 6.00 | 5.00 | 2 | 0 | 22 | 70.5 | 1 | 55 | 3 | 1 | 2 |
| 23 | poptre | Mertens | 1816 | 1816 | LINN | 1546.12 | 451.1 | 6.00 | 3.75 | 5 | 1 | 13 | 68.2 | 1 | 45 | 2 | 1 | 0 |
| 24 | sambuc | Backer | 1928 | 1928 | L | L.2973521 | 582.2 | 3.50 | 2.00 | 2 | 1 | 23 | 76.1 | 1 | 50 | 3 | 3 | 3 |
| 25 | acetat | Saddler | 1826 | 1826 | P | P05300060 | . | 5.50 | 3.50 | 3 | 1 | 13 | . | 1 | 20 | 3 | 0 | 0 |
| 26 | corylu | Fayvush et al. | 2008 | 2008 | NY | 02595412 | 508.3 | 1.50 | 2.50 | 1 | . | 9 | 106.5 | 0 | 30 | 2 | 1 | 0 |
| 27 | betula | . | c.1550 | 1550 | L | . | . | 1.00 | 3.75 | 1 | . | 4 | . | 0 | 10 | 0 | 0 | 0 |
| 28 | querob | Linnaeus (?) | b.1775 | 1775 | S | S09-19031 | 529.7 | 5.50 | 3.75 | 1 | . | 25 | 111.4 | 1 | 60 | 3 | 1 | 1 |
| 29 | poptre | Meinshausen | 1860 | 1860 | MW | MW0300896 | 520.3 | 3.00 | 5.00 | 3 | 0 | 31 | 66.8 | 0 | 50 | 3 | 1 | 0 |
| 30 | acepla | Steinitz | 1882 | 1882 | AMD | AMD.107165 | 539.7 | 5.50 | 6.00 | 6 | 1 | 25 | 90.5 | 1 | 50 | 3 | 1 | 3 |
| 31 | pyrus_ | Odé | 2013 | 2013 | L | L.2072942 | 583.3 | 4.00 | 2.00 | 4 | 1 | 38 | 56.7 | 1 | 35 | 3 | 3 | 0 |
| 32 | pyrus_ | . | 1853 | 1853 | L | L.1904085 | 450.0 | 5.00 | 2.00 | 2 | 0 | 56 | 82.7 | 1 | 30 | 3 | 1 | 1 |
| 33 | acetat | Kitaitsev | 1923 | 1923 | MW | MW0434043 | 528.0 | 3.00 | 3.00 | 3 | 1 | 5 | 78.8 | 1 | 20 | 1 | 0 | 0 |
| 34 | betula | Trautvetter | 1842 | 1842 | LE | LE01051993 | 485.9 | 4.50 | 2.50 | 3 | 1 | 45 | 47.6 | 1 | 40 | 3 | 0 | 0 |
| 35 | tilpla | Brummitt | 1995 | 1995 | NY | 03836631 | 509.4 | 5.50 | 5.00 | 1 | . | 17 | 80.8 | 1 | 40 | 3 | 0 | 0 |
| 36 | acetat | Laxman | b.1796 | 1796 | P | P05215964 | . | 4.00 | 2.50 | 1 | . | 4 | . | 1 | 35 | 2 | 1 | 3 |
| 37 | acepla | Franqueville | 1853 | 1853 | P | P04795066 | . | 5.00 | 2.00 | 2 | 1 | 8 | . | 1 | 30 | 3 | 3 | 2 |
| 38 | acetat | Candolle | b.1824 | 1824 | GDC | G00211418,  G00211419 | 454.6 | 1.50 | 3.50 | 2 | 0 | 17 | 74.6 | 1 | 40 | 3 | 0 | 2 |
| 39 | sambuc | Bourgeau | 1847 | 1847 | P | P03707752 | . | 4.00 | 3.50 | 1 | . | 5 | . | 1 | 30 | 3 | 2 | 0 |
| 40 | acetat | Müller | b.1824 | 1824 | MW | MW0434103 | 526.3 | 5.50 | 1.50 | 5 | 1 | 6 | 93.9 | 1 | 30 | 1 | 1 | 0 |
| 41 | pyrus_ | Theodorov & Fedorov | 1936 | 1936 | LE | LE01010159 | 505.7 | 1.50 | 1.50 | 2 | 1 | 21 | 102.0 | 1 | 50 | 3 | 2 | 1 |
| 42 | sambuc | Camus | 1844 | 1844 | P | P04334263 | . | 5.00 | 5.50 | 1 | . | 4 | . | 1 | 40 | 3 | 0 | 0 |
| 43 | acepla | Wiazemsky | 1866–1869 | 1869 | MW | MW0433743 | 521.2 | 1.33 | 3.67 | 2 | 0 | 4 | 99.5 | 0 | 25 | 2 | 2 | 2 |
| 44 | poptre | Wttewaall | 1833 | 1833 | L | L.3172988 | 560.8 | 3.00 | 3.50 | 1 | . | 29 | 54.7 | 0 | 20 | 2 | 1 | 0 |
| 45 | sambuc | Pocock | 1823 | 1823 | BM | BM001161532 | 546.2 | 3.67 | 5.00 | 1 | . | 2 | 71.6 | 1 | 15 | 3 | 0 | 0 |
| 46 | alnglu | Gowdey | 1966 | 1966 | NY | 02332671 | 509.7 | 5.50 | 5.00 | 1 | . | 17 | 63.4 | 1 | 30 | 3 | 1 | 0 |
| 47 | querob | Dar & Arif | 1976 | 1976 | U | U.1322645 | 525.8 | 3.50 | 5.50 | 2 | 2 | 16 | 101.5 | 0 | 15 | 2 | 0 | 1 |
| 48 | corylu | Boom | 1956 | 1956 | L | L.1559576 | 586.1 | 4.50 | 1.50 | 2 | 1 | 35 | 110.6 | 1 | 60 | 3 | 1 | 3 |
| 49 | betula | Vaillant | b.1722 | 1722 | P | P00530008 | . | 4.25 | 2.50 | 1 | . | 70 | . | 1 | 25 | 3 | 1 | 0 |
| 50 | tilpla | Bouby | 1973 | 1973 | P | P00685220 | . | 5.50 | 5.00 | 1 | . | 8 | . | 1 | 40 | 3 | 0 | 0 |
| 51 | poptre | Stolyarskaya | 1985 | 1985 | MW | MW0300884 | 531.4 | 4.00 | 1.50 | 2 | 0 | 72 | 55.1 | 0 | 40 | 3 | 0 | 0 |
| 52 | poptre | Sande Lacoste | 1841 | 1841 | L | L.3172998 | 585.6 | 3.00 | 4.00 | 1 | . | 47 | 30.6 | 0 | 20 | 3 | 1 | 0 |
| 53 | alnglu | Naczi | 2009 | 2009 | NY | 02342519 | 510.2 | 5.50 | 6.00 | 5 | 1 | 5 | 99.6 | 1 | 40 | 2 | 0 | 0 |
| 54 | acetat | Smirnova | 1984 | 1984 | NY | 02591663 | 506.7 | 5.50 | 6.00 | 2 | 0 | 20 | 88.7 | 1 | 40 | 2 | 1 | 1 |
| 55 | poptre | Kotschy | 1859 | 1859 | P | P00741341 | 504.3 | 4.50 | 3.00 | 2 | 0 | 23 | 53.4 | 0 | 40 | 2 | 1 | 1 |
| 56 | corylu | Yakovlev | 1995 | 1995 | MW | MW0049859 | 496.2 | 4.00 | 2.00 | 4 | 1 | 16 | 104.5 | 1 | 60 | 2 | 0 | 0 |
| 57 | betula | Montretor (?) | 1885 | 1885 | LE | LE01051996 | 502.3 | 2.00 | 4.50 | 1 | . | 35 | 68.6 | 0 | 40 | 3 | 1 | 1 |
| 58 | corylu | Zuccarini | 1843 | 1843 | K | K000859905 | 485.5 | 3.00 | 3.50 | 2 | 2 | 4 | 101.6 | 0 | 40 | 0 | 0 | 1 |
| 59 | tilpla | Cosson | 1835 | 1835 | P | P06700723 | . | 5.00 | 1.50 | 1 | . | 21 | . | 1 | 70 | 3 | 1 | 1 |
| 60 | alnglu | Requien | 1850 | 1850 | P | P00536972 | . | 2.00 | 4.00 | 2 | 1 | 15 | . | 1 | 20 | 2 | 1 | 1 |
| 61 | corylu | Hummel | 1930 | 1930 | S | S-GH-981 | 515.4 | 4.50 | 5.50 | 2 | 0 | 13 | 70.0 | 1 | 40 | 1 | 1 | 0 |
| 62 | sambuc | Martet | 1807 | 1807 | P | P03707277 | . | 4.00 | 5.75 | 1 | . | 2 | . | 1 | 15 | 2 | 1 | 0 |
| 63 | querob | . | b.1774 | 1774 | LINN | 1128.30 | 359.2 | 4.25 | 5.50 | 2 | 0 | 1 | 83.3 | 0 | 70 | 2 | 0 | 0 |
| 64 | corylu | Hers | 1923 | 1923 | P | P06811361 | . | 2.50 | 4.50 | 1 | . | 5 | . | 1 | 40 | 2 | 1 | 0 |
| 65 | alnglu | Ronniger | 1919 | 1919 | NY | 03500325 | 513.9 | 4.50 | 6.00 | 1 | . | 10 | 75.9 | 1 | 20 | 3 | 1 | 0 |
| 66 | alnglu | Tikhomirov & Nikonorova | 1977 | 1977 | MW | MW0314376 | 525.5 | 2.00 | 2.00 | 1 | . | 24 | 98.8 | 0 | 70 | 3 | 1 | 0 |
| 67 | acepla | Morong | 1876 | 1876 | NY | 02487535 | 481.8 | 4.50 | 2.50 | 2 | 0 | 8 | 106.9 | 1 | 35 | 1 | 0 | 0 |
| 68 | corylu | Palmer | 1946 | 1946 | WAG | WAG.1334358 | 492.6 | 5.00 | 4.00 | 1 | . | 18 | 99.8 | 1 | 50 | 3 | 1 | 0 |
| 69 | corylu | Buser | 1903 | 1903 | P | P06809904 | . | 3.50 | 2.50 | 3 | 1 | 11 | . | 1 | 60 | 3 | 1 | 1 |
| 71 | tilpla | Racs & Kovacs | 2008 | 2008 | NY | 02629407 | . | 5.50 | 4.00 | 1 | . | 13 | . | 1 | 40 | 3 | 1 | 0 |
| 72 | acetat | Karakash | 1949 | 1949 | MW | MW0434189 | 537.6 | 3.50 | 1.50 | 1 | . | 70 | 50.6 | 1 | 40 | 3 | 1 | 2 |
| 73 | alnglu | Canby | 1863 | 1863 | NY | 02471642 | . | 4.00 | 2.50 | 1 | . | 12 | . | 1 | 20 | 3 | 1 | 0 |
| 74 | alnglu | Stainton & Henderson | 1960 | 1960 | E | E00240370 | 498.2 | 5.50 | 5.00 | 1 | . | 44 | 44.6 | 1 | 50 | 3 | 1 | 0 |
| 75 | poptre | Kozhin | 2011 | 2011 | MW | MW0300862 | 505.8 | 4.00 | 1.50 | 2 | 2 | 43 | 58.6 | 0 | 60 | 3 | 0 | 0 |
| 76 | sambuc | Staring | 1832 | 1832 | WAG | WAG.1506258 | 495.2 | 2.00 | 5.00 | 2 | 1 | 1 | 115.0 | 1 | 25 | 3 | 0 | 0 |
| 77 | pyrus_ | Pospelova | 1975 | 1975 | MW | MW0097698 | 524.3 | 3.50 | 1.00 | 1 | . | 23 | 63.9 | 0 | 30 | 3 | 1 | 0 |
| 78 | pyrus_ | Maack | 1859 | 1859 | P | P01819373 | 478.1 | 3.00 | 2.00 | 3 | 2 | 23 | 102.1 | 0 | 60 | 3 | 1 | 0 |
| 79 | querob | Nee | 2012 | 2012 | NY | 01801206 | 495.6 | 6.00 | 3.50 | 2 | 2 | 33 | 118.7 | 1 | 50 | 3 | 1 | 0 |
| 80 | poptre | Sherard | b.1728 | 1728 | OXF | . | 427.4 | 3.33 | 3.67 | 1 | . | 42 | 36.7 | 0 | 40 | 3 | 1 | 0 |
| 81 | querob | Commons | 1896 | 1896 | NY | 01465817 | . | 5.50 | 3.00 | 1 | . | 30 | . | 1 | 30 | 3 | 0 | 0 |
| 82 | sambuc | Linné fil. | b.1783 | 1783 | S | S09-33862 | 377.7 | 4.50 | 4.00 | 1 | . | 4 | 98.3 | 1 | 40 | 2 | 1 | 0 |
| 83 | querob | Nasarow | 1913–1914 | 1914 | MW | MW0315068 | 582.5 | 5.50 | 2.50 | 4 | 1 | 21 | 116.4 | 1 | 35 | 3 | 1 | 1 |
| 84 | querob | Vaillant | b.1722 | 1722 | P | P06857404 | . | 3.50 | 4.00 | 1 | . | 56 | . | 1 | 40 | 3 | 1 | 0 |
| 85 | betula | Vialles (?) | 1813 | 1813 | MW | MW0313945 | 512.8 | 5.50 | 3.00 | 3 | 1 | 50 | 47.1 | 1 | 40 | 3 | 1 | 0 |
| 86 | alnglu | . | c.1550 | 1550 | L | . | . | 3.00 | 3.80 | 1 | . | 8 | . | 1 | 25 | 3 | 1 | 0 |
| 87 | poptre | Guinier | 1939 | 1939 | P | P05599937 | . | 4.50 | 3.50 | 2 | 2 | 21 | . | 0 | 60 | 3 | 0 | 0 |
| 88 | tilpla | van Royen | 1979 | 1979 | L | L.2361495 | 583.1 | 3.00 | 4.00 | 1 | . | 16 | 61.7 | 1 | 30 | 3 | 1 | 1 |
| 89 | betula | Goldbach | b.1824 | 1824 | MW | MW0770868 | 526.8 | 3.50 | 3.00 | 1 | . | 10 | 69.0 | 0 | 20 | 1 | 0 | 0 |
| 90 | sambuc | Vaillant | b.1722 | 1722 | P | P03707197 | . | 4.25 | 4.25 | 1 | . | 4 | . | 1 | 35 | 3 | 0 | 0 |
| 91 | poptre | Clifford | b.1760 | 1760 | BM | BM000647500 | 457.5 | 2.00 | 5.00 | 1 | . | 11 | 51.3 | 0 | 20 | 0 | 0 | 0 |
| 92 | corylu | Silva | 1946 | 1946 | P | P06809900 | . | 4.00 | 5.50 | 1 | . | 15 | . | 1 | 45 | 2 | 1 | 0 |
| 93 | alnglu | Sintenis | 1883 | 1883 | P | P00539102 | . | 5.50 | 6.00 | 3 | 1 | 31 | . | 1 | 50 | 3 | 1 | 1 |
| 94 | tilpla | Scheppig | 1855 | 1855 | AMD | AMD.89915 | 514.3 | 2.00 | 3.00 | 2 | 0 | 13 | 88.0 | 1 | 60 | 2 | 0 | 1 |
| 95 | betula | Goroschankin | 1869 | 1869 | MW | MW0313473 | 425.9 | 5.00 | 4.00 | 4 | 1 | 24 | 60.8 | 1 | 30 | 2 | 1 | 0 |
| 96 | sambuc | Westhoff | 1932 | 1932 | U | U.1056594 | 496.7 | 2.00 | 3.50 | 2 | 1 | 4 | 111.9 | 1 | 25 | 3 | 1 | 0 |
| 97 | pyrus_ | Davis | 1954 | 1954 | E | E00408484 | 489.4 | 2.00 | 2.00 | 1 | . | 16 | 57.9 | 0 | 20 | 2 | 1 | 0 |
| 98 | sambuc | Steinitz | 1881 | 1881 | AMD | AMD.24650 | 536.8 | 4.00 | 2.50 | 3 | 1 | 5 | 121.5 | 1 | 40 | 3 | 1 | 1 |
| 100 | alnglu | . | 1821 | 1821 | P | P06746494 | . | 4.67 | 3.33 | 1 | . | 11 | . | 1 | 30 | 2 | 0 | 1 |
| 102 | tilpla | Billot | 1847 | 1847 | WAG | WAG.1856854 | 463.1 | 3.00 | 3.00 | 1 | . | 3 | 72.2 | 1 | 20 | 2 | 0 | 1 |
| 103 | poptre | Schreiner | 1924 | 1924 | NY | 02470800 | 514.6 | 5.00 | 5.50 | 3 | 0 | 36 | 56.1 | 0 | 40 | 3 | 1 | 0 |
| 104 | sambuc | Scheppig | 1891 | 1891 | AMD | AMD.24649 | 540.8 | 5.00 | 3.50 | 1 | . | 5 | 117.0 | 1 | 30 | 3 | 1 | 2 |
| 105 | betula | Vaillant | 1702 | 1702 | P | P00530009 | . | 5.33 | 3.00 | 1 | . | 34 | . | 1 | 25 | 3 | 1 | 0 |
| 106 | pyrus_ | Pourret | b.1818 | 1818 | P | P03141036 | . | 1.70 | 4.30 | 3 | 0 | 10 | . | 1 | 35 | 2 | 2 | 0 |
| 107 | tilpla | Vincent | 1847 | 1847 | L | L.2361701 | 528.0 | 4.50 | 4.50 | 3 | 0 | 17 | 85.5 | 1 | 50 | 2 | 1 | 1 |
| 108 | sambuc | Vaillant | b.1722 | 1722 | P | P03707753 | . | 4.50 | 4.00 | 2 | 0 | 3 | . | 1 | 40 | 3 | 0 | 1 |
| 109 | alnglu | Sherard | b.1728 | 1728 | OXF | . | 421.1 | 4.40 | 5.60 | 1 | . | 9 | 78.6 | 1 | 50 | 1 | 0 | 0 |
| 110 | acetat | Sintenis | 1890 | 1890 | P | P05300133 | . | 4.50 | 1.00 | 2 | 1 | 39 | . | 1 | 40 | 3 | 2 | 1 |
| 111 | querob | Craig-Christie | 1887 | 1887 | E | E00841682 | 538.2 | 6.00 | 2.50 | 2 | 0 | 60 | 79.1 | 1 | 75 | 3 | 1 | 1 |
| 112 | tilpla | Schmidt | 1982 | 1982 | BASBG | BASBG-00005187 | 493.0 | 4.00 | 5.50 | 1 | . | 7 | 88.2 | 1 | 40 | 3 | 0 | 1 |
| 113 | alnglu | Shvedchikova | 2001 | 2001 | MW | MW314343 | 504.1 | 2.50 | 6.00 | 1 | . | 13 | 75.2 | 0 | 40 | 3 | 1 | 0 |
| 114 | pyrus_ | Tobey | 1965 | 1965 | E | E00408581 | 492.7 | 2.50 | 4.00 | 2 | 1 | 47 | 37.9 | 0 | 25 | 3 | 1 | 0 |
| 115 | tilpla | Vaillant | b.1722 | 1722 | P | P06702465 | . | 1.33 | 3.00 | 1 | . | 5 | . | 0 | 40 | 3 | 0 | 0 |
| 116 | corylu | Valckenier Suringar | 1908 | 1908 | WAG | WAG.1334355 | 498.3 | 2.50 | 6.00 | 2 | 2 | 7 | 89.5 | 0 | 45 | 2 | 3 | 2 |
| 117 | tilpla | Renaud | 1923 | 1923 | P | P00983565 | 354.5 | 3.50 | 4.00 | 1 | . | 5 | 82.1 | 1 | 20 | 3 | 0 | 1 |
| 118 | betula | Maslakov | 1960 | 1960 | LE | LE01053002 | 518.8 | 2.00 | 5.00 | 3 | 1 | 55 | 54.8 | 1 | 40 | 3 | 1 | 0 |
| 119 | acetat | Shuvarikov | 1982 | 1982 | MW | MW0434038 | 534.7 | 4.50 | 2.00 | 2 | 0 | 14 | 80.2 | 1 | 35 | 2 | 1 | 1 |
| 120 | acepla | Kobus | 1879 | 1879 | L | L.0172943 | 586.7 | 1.50 | 3.00 | 1 | . | 9 | 171.4 | 0 | 40 | 3 | 1 | 1 |
| 121 | querob | Jovet | 1991 | 1991 | P | P04185278 | 486.7 | 3.00 | 4.50 | 2 | 0 | 22 | 111.0 | 0 | 40 | 3 | 1 | 1 |
| 122 | poptre | van Nek | 1992 | 1992 | WAG | WAG.1210894 | 486.1 | 2.50 | 3.50 | 1 | . | 57 | 34.9 | 0 | 40 | 3 | 1 | 0 |
| 123 | poptre | Baenitz | 1894 | 1894 | P | P05599924 | . | 5.50 | 1.50 | 4 | 1 | 25 | . | 1 | 45 | 3 | 0 | 0 |
| 124 | tilpla | Brongniart | 1823 | 1823 | P | P06702477 | . | 3.80 | 3.20 | 2 | 1 | 12 | . | 1 | 60 | 3 | 1 | 0 |
| 125 | acetat | Partsch | 1819 | 1819 | P | P05215963 | 532.5 | 4.00 | 5.50 | 5 | 1 | 11 | 89.4 | 1 | 30 | 1 | 0 | 1 |
| 126 | betula | Byalt et al. | 2016 | 2016 | LE | LE01051992 | 505.9 | 4.00 | 6.00 | 1 | . | 32 | 45.9 | 0 | 25 | 3 | 1 | 0 |
| 127 | querob | Sperling et al. | 1985 | 1985 | E | E00401689 | 517.5 | 5.50 | 5.00 | 1 | . | 16 | 129.6 | 1 | 35 | 3 | 1 | 1 |
| 128 | betula | Craig-Christie | 1878 | 1878 | E | E00773274 | 492.1 | 3.50 | 3.50 | 2 | 0 | 35 | 54.2 | 0 | 40 | 3 | 0 | 0 |
| 129 | tilpla | Kramer | 1976 | 1976 | U | U.1377722 | 594.4 | 5.50 | 3.00 | 1 | . | 8 | 129.4 | 1 | 40 | 3 | 1 | 1 |
| 130 | querob | Tenore | b.1861 | 1861 | BM | BM000613043 | . | 3.00 | 5.00 | 1 | . | 15 | . | 0 | 35 | 3 | 0 | 1 |
| 131 | acepla | Bladlok | 1904 | 1904 | U | U.1579470 | 566.2 | 5.00 | 3.50 | 2 | 1 | 19 | 96.2 | 1 | 25 | 3 | 1 | 2 |
| 132 | sambuc | Henning | 1796 | 1796 | MW | MW0522467 | 531.1 | 4.33 | 4.67 | 1 | . | 4 | 142.9 | 1 | 20 | 3 | 1 | 0 |
| 133 | betula | Bieberstein | 1820 | 1820 | LE | LE01051994 | 453.3 | 5.50 | 6.00 | 2 | 0 | 32 | 66.3 | 1 | 25 | 3 | 1 | 0 |
| 134 | sambuc | Troupin | 1972 | 1972 | WAG | WAG.1506302 | 493.8 | 2.50 | 3.00 | 2 | 2 | 8 | 140.2 | 1 | 50 | 3 | 3 | 0 |
| 135 | tilpla | Stohl | b.1896 | 1896 | JE | JE00019534 | 510.0 | 5.00 | 2.00 | 2 | 1 | 21 | 69.8 | 1 | 60 | 3 | 1 | 1 |
| 136 | corylu | Hartweg | 1848 | 1848 | K | K000859889 | 491.5 | 4.50 | 3.50 | 1 | . | 12 | 74.3 | 1 | 35 | 2 | 3 | 2 |
| 137 | acepla | Brumbach | 1974 | 1974 | NY | 02487566 | 505.8 | 5.00 | 4.50 | 1 | . | 8 | 113.2 | 1 | 50 | 3 | 1 | 1 |
| 138 | pyrus_ | Boer | 1864 | 1864 | L | L.1904086 | . | 2.50 | 4.50 | 1 | . | 35 | . | 0 | 30 | 3 | 2 | 0 |
| 139 | acetat | Sag | 1986 | 1986 | P | P04795542 | . | 6.00 | 5.50 | 2 | 0 | 35 | . | 1 | 70 | 3 | 1 | 2 |
| 140 | poptre | Sag | 1981 | 1981 | P | P00036563 | . | 4.00 | 4.00 | 1 | . | 23 | . | 0 | 45 | 3 | 0 | 0 |
| 141 | sambuc | Wallich | 1818 | 1818 | BM | BM000521767 | 520.1 | 3.00 | 4.33 | 6 | 0 | 12 | 207.1 | 1 | 45 | 3 | 3 | 3 |
| 142 | poptre | Nyman | 1858 | 1858 | P | P05599926 | . | 6.00 | 4.00 | 2 | 1 | 20 | . | 1 | 20 | 2 | 0 | 0 |
| 143 | pyrus_ | Wright | 1853–1856 | 1856 | K | K000758079 | 473.8 | . | . | 1 | . | 13 | 78.5 | 1 | 40 | 3 | 1 | 0 |
| 144 | acetat | Boschnjak | b.1831 | 1831 | MW | MW0434274 | 534.9 | 5.50 | 4.50 | 2 | 0 | 28 | 53.1 | 1 | 50 | 1 | 2 | 1 |
| 145 | acepla | Sherard | b.1728 | 1728 | OXF | . | 444.9 | 2.00 | 3.00 | 1 | . | 7 | 116.1 | 0 | 60 | 3 | 3 | 2 |
| 146 | querob | Makarov | 1965 | 1965 | MHA | MHA0047088 | 536.3 | 5.00 | 4.00 | 1 | . | 43 | 98.0 | 1 | 60 | 3 | 1 | 0 |
| 147 | querob | Stojanov et al. | 1952 | 1952 | MW | MW0771214 | 493.9 | 4.50 | 5.50 | 1 | . | 21 | 98.3 | 1 | 30 | 3 | 1 | 0 |
| 148 | tilpla | Yuncker | 1942 | 1942 | NY | 02557496 | 512.4 | 5.00 | 4.50 | 1 | . | 10 | 84.1 | 1 | 40 | 3 | 0 | 1 |
| 149 | betula | Shukhobodsky | 1975 | 1975 | L | L.1558868 | 585.8 | 2.50 | 4.00 | 6 | 2 | 12 | 82.5 | 0 | 25 | 1 | 0 | 0 |
| 150 | pyrus_ | Gravet | 1879 | 1879 | L | L.1904092 | 571.1 | 3.50 | 3.00 | 1 | . | 33 | 29.5 | 0 | 15 | 3 | 1 | 0 |
| 151 | tilpla | Schouten | 1956 | 1956 | U | U.1377723 | 595.8 | 4.00 | 2.00 | 2 | 0 | 13 | 82.5 | 1 | 40 | 3 | 0 | 0 |
| 152 | pyrus_ | Moll | 1897 | 1897 | WAG | WAG.1886867 | 484.3 | 5.00 | 3.00 | 4 | 0 | 82 | 40.3 | 1 | 50 | 3 | 3 | 3 |
| 153 | querob | Senoner | 1832 | 1832 | MW | MW0771205 | 495.2 | 3.00 | 2.00 | 1 | . | 17 | 122.5 | 0 | 30 | 3 | 1 | 0 |
| 154 | tilpla | Sherard | b.1728 | 1728 | OXF | . | 432.4 | 4.50 | 5.50 | 1 | . | 5 | 82.7 | 1 | 40 | 2 | 0 | 0 |
| 155 | acetat | Barta | 2008 | 2008 | NY | 03082343 | 510.1 | 5.50 | 4.50 | 3 | 0 | 24 | 111.5 | 1 | 70 | 2 | 1 | 1 |
| 156 | pyrus_ | Wiemann | 1903 | 1903 | L | L.1904034 | 537.8 | 5.00 | 2.50 | 3 | 1 | 71 | 51.3 | 1 | 40 | 3 | 3 | 0 |
| 157 | alnglu | Maksymovych | 1824–1826 | 1826 | MW | MW0314446 | 514.5 | 3.00 | 2.50 | 1 | . | 8 | 45.7 | 1 | 15 | 3 | 1 | 1 |
| 158 | betula | Güner et al. | 2005 | 2005 | E | E00210475 | 493.6 | 4.50 | 1.00 | 2 | 1 | 6 | 94.2 | 1 | 25 | 2 | 3 | 0 |
| 159 | pyrus_ | Seregin | 2004 | 2004 | MW | MW0384772 | 513.6 | 5.00 | 6.00 | 1 | . | 34 | 55.6 | 0 | 35 | 3 | 2 | 1 |
| 160 | poptre | Kozlowsky | 1913 | 1913 | E | E00448546 | 504.9 | 5.00 | 3.50 | 3 | 1 | 17 | 71.4 | 1 | 40 | 3 | 1 | 1 |
| 161 | acepla | Pteancu | 1943 | 1943 | P | P04765382 | . | 3.50 | 2.00 | 1 | . | 5 | . | 1 | 20 | 3 | 1 | 0 |
| 162 | betula | Groep | 1971 | 1971 | L | L.1558865 | 572.2 | 5.50 | 2.00 | 1 | . | 68 | 45.4 | 1 | 35 | 3 | 1 | 1 |
| 163 | poptre | Sherard | b.1728 | 1728 | OXF | . | 396.2 | 1.00 | 2.00 | 2 | 2 | 5 | 51.6 | 0 | 15 | 3 | 0 | 0 |
| 164 | corylu | Poiret | b.1825 | 1825 | P | P06810653 | . | 2.00 | 4.40 | 2 | 1 | 6 | . | 1 | 40 | 0 | 0 | 1 |
| 165 | acepla | . | c.1550 | 1550 | L | . | . | 1.75 | 2.00 | 1 | . | 1 | . | 0 | 50 | 0 | 1 | 1 |
| 166 | pyrus_ | . | 1887 | 1887 | P | P03132061 | . | 3.50 | 3.50 | 1 | . | 18 | . | 1 | 20 | 3 | 2 | 0 |
| 167 | poptre | Braun | 1845 | 1845 | K | K000592100 | 354.7 | 2.50 | 3.50 | 3 | 0 | 13 | 51.5 | 1 | 40 | 2 | 1 | 0 |
| 168 | acetat | Manissadjian | 1893 | 1893 | E | E00436962 | 494.2 | 5.00 | 4.50 | 3 | 0 | 7 | 84.6 | 1 | 50 | 2 | 0 | 2 |
| 169 | corylu | Poiret | b.1825 | 1825 | P | P06810660 | . | 2.60 | 4.40 | 1 | . | 12 | . | 0 | 45 | 2 | 0 | 1 |
| 170 | tilpla | Hippe | 1877 | 1877 | K | K000687785,  K000687786 | 415.0 | 5.00 | 3.50 | 3 | 1 | 10 | 72.6 | 1 | 50 | 2 | 0 | 1 |
| 171 | querob | . | 1839 | 1839 | E | E00841681 | 502.7 | 1.50 | 4.50 | 1 | . | 17 | 91.0 | 0 | 60 | 3 | 0 | 0 |
| 172 | corylu | Marcilly | 1845 | 1845 | P | P06809821 | . | 4.00 | 3.50 | 2 | 1 | 5 | . | 1 | 40 | 0 | 0 | 0 |
| 173 | sambuc | Borgsteede | 1967 | 1967 | AMD | AMD.24647 | 527.1 | 4.50 | 3.50 | 2 | 0 | 12 | 162.9 | 1 | 50 | 3 | 1 | 2 |
| 174 | betula | Maximowicz | 1855 | 1855 | LE | LE01016926 | 492.2 | 5.00 | 4.00 | 2 | 0 | 34 | 60.0 | 1 | 25 | 3 | 1 | 1 |
| 175 | alnglu | Rostan | 1880 | 1880 | L | L.1560831 | 565.9 | 1.00 | 3.50 | 5 | 0 | 10 | 131.4 | 1 | 45 | 1 | 2 | 1 |
| 176 | corylu | Clifford | b.1753 | 1753 | BM | BM000647428 | 509.6 | 5.00 | 2.50 | 2 | 1 | 7 | 134.1 | 1 | 50 | 1 | 0 | 0 |
| 177 | betula | Marshall | 1916 | 1916 | E | E00773220 | 493.6 | 1.50 | 2.50 | 4 | 0 | 70 | 30.2 | 0 | 40 | 2 | 1 | 0 |
| 178 | alnglu | Meschayeff | 1868 | 1868 | MW | MW0314383 | 585.3 | 2.00 | 4.50 | 3 | 0 | 25 | 61.4 | 0 | 40 | 2 | 1 | 1 |
| 179 | betula | Thedenius | 1836 | 1836 | P | P00532820 | . | 4.50 | 2.50 | 2 | 0 | 44 | . | 1 | 30 | 3 | 0 | 0 |
| 180 | acepla | Gillett | 1939 | 1939 | NY | 02487603 | 515.5 | 3.50 | 2.50 | 5 | 1 | 7 | 108.4 | 1 | 40 | 3 | 0 | 0 |
| 181 | acetat | Zinger & Koschewnikoff | 1878 | 1878 | MW | MW0434037 | 517.6 | 1.50 | 4.00 | 2 | 0 | 16 | 88.0 | 1 | 35 | 2 | 3 | 1 |
| 182 | corylu | Boccone (?) | c.1671 | 1671 | P | P06810662 | . | 2.00 | 2.00 | 2 | 0 | 36 | . | 0 | 70 | 3 | 0 | 0 |
| 183 | alnglu | Boschnjak | 1826 | 1826 | MW | MW0314356 | 512.2 | 1.00 | 3.50 | 1 | . | 8 | 72.1 | 0 | 20 | 3 | 0 | 0 |
| 184 | poptre | Clifford | b.1760 | 1760 | BM | BM000647499 | 480.0 | 4.00 | 4.67 | 1 | . | 43 | 38.1 | 0 | 45 | 2 | 0 | 0 |
| 185 | alnglu | Jalicon | 1934 | 1934 | P | P00536932 | . | 4.50 | 3.00 | 5 | 1 | 23 | . | 1 | 40 | 3 | 2 | 2 |
| 186 | corylu | Henry | 1887 | 1887 | K | K000859900 | 476.9 | 3.50 | 2.00 | 2 | 1 | 13 | 80.6 | 1 | 50 | 1 | 3 | 3 |
| 187 | alnglu | Kelberer | 1925 | 1925 | MW | MW0314367 | 508.7 | 2.00 | 3.50 | 2 | 0 | 14 | 96.8 | 0 | 30 | 2 | 0 | 2 |
| 188 | querob | Mathieu & Billot | 1858 | 1858 | WAG | WAG.1318652 | 583.3 | 5.00 | 1.50 | 5 | 1 | 40 | 98.9 | 1 | 40 | 2 | 1 | 0 |
| 189 | acepla | Spach | 1832 | 1832 | P | P04794741 | . | 4.00 | 3.00 | 3 | 0 | 11 | . | 1 | 50 | 3 | 1 | 2 |
| 190 | querob | Schur | 1870 | 1870 | P | P06856885 | 505.2 | 3.50 | 2.50 | 1 | . | 25 | 114.6 | 1 | 60 | 3 | 1 | 0 |
| 191 | betula | Kramer | 1985 | 1985 | U | U.1144097 | 595.3 | 6.00 | 5.00 | 1 | . | 30 | 53.6 | 1 | 25 | 3 | 1 | 0 |
| 192 | acetat | Krylov & Esipov | 2008 | 2008 | MW | MW0434047 | 510.0 | 2.50 | 3.00 | 2 | 0 | 23 | 111.2 | 0 | 75 | 3 | 1 | 0 |
| 193 | acepla | Ehrhart | b.1795 | 1795 | LINN | 1582.12 | 437.0 | 2.25 | 3.75 | 1 | . | 4 | 83.7 | 0 | 20 | 2 | 0 | 0 |
| 194 | alnglu | Schrenk | 1868 | 1868 | P | P00536995 | 520.5 | 4.00 | 3.00 | 4 | 1 | 19 | 67.2 | 1 | 25 | 3 | 2 | 3 |
| 195 | acetat | Candolle | b.1824 | 1824 | GDC | G00211372,  G00211444,  G00211445 | 482.1 | 3.50 | 2.50 | 3 | 0 | 20 | 82.0 | 1 | 60 | 2 | 1 | 1 |
| 196 | betula | Erikson | 1926 | 1926 | L | L.1558867 | 587.2 | 1.50 | 2.00 | 4 | 0 | 20 | 51.4 | 0 | 25 | 3 | 1 | 1 |
| 197 | tilpla | van Hattum | 1968 | 1968 | L | L.3703058 | 580.6 | 1.00 | 5.00 | 1 | . | 6 | 126.9 | 0 | 30 | 2 | 0 | 1 |
| 198 | acepla | Velthuis | 1983 | 1983 | WAG | WAG.1405174 | 502.4 | 1.50 | 6.00 | 1 | . | 5 | 109.5 | 0 | 50 | 3 | 1 | 0 |
| 199 | tilpla | Berger | 1967 | 1967 | BASBG | BASBG-00005192 | 494.8 | 1.50 | 1.00 | 1 | . | 50 | 72.7 | 0 | 60 | 3 | 2 | 2 |
| 200 | alnglu | Yanushevsky | 1951 | 1951 | MW | MW0314345 | 530.3 | 1.50 | 1.50 | 4 | 0 | 32 | 61.9 | 0 | 40 | 3 | 1 | 0 |
| 201 | poptre | Katz & Machotina | 1925 | 1925 | MW | MW0300961 | 533.0 | 5.50 | 4.00 | 2 | 0 | 13 | 69.1 | 0 | 50 | 2 | 0 | 0 |
| 202 | tilpla | Wierzbicki | 1840 | 1840 | L | L.2360995 | 583.3 | 4.50 | 1.50 | 2 | 1 | 42 | 72.5 | 1 | 80 | 3 | 1 | 1 |
| 203 | acepla | Danielsen | 1969 | 1969 | L | L.0172913 | 583.9 | 5.00 | 3.00 | 1 | . | 8 | 121.1 | 1 | 30 | 3 | 1 | 0 |
| 204 | corylu | Groult | 1996 | 1996 | P | P06809989 | . | 1.00 | 4.00 | 2 | 2 | 8 | . | 0 | 20 | 2 | 1 | 0 |
| 205 | acetat | Henning | b.1838 | 1838 | MW | MW0434082 | 542.8 | 3.50 | 4.50 | 1 | . | 1 | 62.2 | 1 | 15 | 1 | 0 | 2 |
| 206 | betula | Camus | 1866 | 1866 | P | P00528839 | . | 4.00 | 4.50 | 1 | . | 37 | . | 1 | 25 | 3 | 0 | 0 |
| 207 | corylu | Candolle | b.1822 | 1822 | P | P06810707 | . | 4.80 | 3.60 | 2 | 1 | 4 | . | 1 | 40 | 0 | 0 | 0 |
| 208 | querob | Kotschy | 1853 | 1853 | K | K000832030 | 537.8 | 1.50 | 2.50 | 5 | 1 | 34 | 115.0 | 1 | 60 | 3 | 2 | 2 |
| 209 | pyrus_ | Alexeev | 1966 | 1966 | MW | MW0677997 | 542.4 | 2.00 | 2.50 | 1 | . | 5 | 48.3 | 1 | 20 | 3 | 1 | 0 |
| 210 | querob | Smith | b.1802 | 1802 | LINN | 1478.19 | . | 3.67 | 4.67 | 4 | 1 | 50 | . | 1 | 70 | 3 | 2 | 1 |
| 211 | corylu | Kaden | 1959 | 1959 | MW | MW0735837 | 534.8 | 4.50 | 2.00 | 9 | 1 | 13 | 83.6 | 1 | 40 | 1 | 3 | 2 |
| 212 | acepla | van Hattum | 1942 | 1942 | L | L.3702215 | 573.8 | 3.50 | 3.00 | 1 | . | 8 | 134.3 | 1 | 25 | 3 | 1 | 1 |
| 213 | pyrus_ | Alexeev | 2001 | 2001 | MW | MW0384776 | 498.9 | 5.00 | 5.50 | 1 | . | 18 | 55.5 | 1 | 25 | 3 | 1 | 1 |
| 214 | acetat | Lanyi | 1915 | 1915 | L | L.2295940 | 552.6 | 3.00 | 5.00 | 2 | 0 | 26 | 84.2 | 1 | 45 | 3 | 1 | 1 |
| 216 | corylu | Spellenberg & Mahrt | 1992 | 1992 | NY | 02478592 | 507.9 | 5.00 | 5.00 | 2 | 1 | 18 | 95.5 | 1 | 40 | 1 | 1 | 2 |
| 217 | acetat | Vrabilyi | 1866 | 1866 | P | P05215965 | 502.7 | 1.00 | 2.00 | 1 | . | 17 | 83.6 | 0 | 20 | 3 | 1 | 1 |
| 218 | betula | Gunnarsson | 1919 | 1919 | S | S16-57070 | 537.7 | 1.50 | 4.00 | 2 | 1 | 39 | 52.6 | 1 | 30 | 3 | 2 | 1 |
| 219 | corylu | David | 1864 | 1864 | P | P06809379 | . | 2.50 | 2.50 | 2 | 0 | 6 | . | 1 | 40 | 2 | 2 | 0 |
| 220 | alnglu | Vaillant | b.1722 | 1722 | P | P00536916 | . | 5.40 | 3.40 | 2 | 1 | 26 | . | 1 | 45 | 2 | 0 | 1 |
| 221 | acetat | Borza | 1920 | 1920 | P | P06602516 | . | 1.00 | 3.50 | 1 | . | 24 | . | 1 | 45 | 3 | 2 | 1 |
| 222 | sambuc | Buysman | 1886 | 1886 | U | U.1056392 | 596.4 | 5.50 | 4.50 | 2 | 0 | 4 | 113.3 | 1 | 45 | 3 | 1 | 0 |
| 223 | corylu | Delavay | 1889 | 1889 | P | P01903229 | 498.0 | 3.00 | 5.50 | 2 | 1 | 17 | 94.0 | 1 | 40 | 3 | 2 | 2 |
| 224 | poptre | Protasova | 1958 | 1958 | MW | MW0300874 | 535.7 | 2.00 | 3.00 | 2 | 0 | 66 | 55.6 | 0 | 50 | 3 | 1 | 0 |
| 225 | tilpla | Toepffer | 1879 | 1879 | AMD | AMD.89943 | 518.3 | 4.00 | 3.00 | 3 | 1 | 5 | 115.4 | 1 | 50 | 3 | 1 | 1 |
| 226 | pyrus_ | Maximowicz | 1856 | 1856 | LE | LE01009639 | 524.6 | 2.00 | 3.00 | 3 | 0 | 36 | 78.3 | 0 | 30 | 3 | 1 | 1 |
| 227 | acepla | Sperling et al. | 1985 | 1985 | E | E00436932 | 496.4 | 6.00 | 5.50 | 1 | . | 7 | 74.6 | 1 | 25 | 2 | 0 | 0 |
| 228 | acepla | Schur | b.1854 | 1854 | L | L.0172949 | 583.3 | 1.50 | 3.50 | 1 | . | 1 | 137.5 | 0 | 15 | 0 | 2 | 1 |
| 229 | tilpla | Zika | 2000 | 2000 | NY | 02557509 | 513.3 | 1.50 | 5.50 | 1 | . | 6 | 106.9 | 0 | 30 | 2 | 0 | 0 |
| 230 | betula | Čuba | 1987 | 1987 | NY | 02477382 | 511.6 | 6.00 | 6.00 | 2 | 0 | 61 | 44.4 | 1 | 30 | 3 | 1 | 0 |
| 231 | sambuc | Toetenel | 2013 | 2013 | L | L.2072725 | 585.3 | 2.50 | 3.00 | 1 | . | 17 | 71.9 | 1 | 20 | 3 | 3 | 0 |
| 232 | tilpla | Buysman | 1889 | 1889 | U | U.1377732 | 596.1 | 4.50 | 5.00 | 1 | . | 7 | 98.9 | 1 | 30 | 3 | 1 | 1 |
| 233 | sambuc | Borchman | 1840 | 1840 | MW | MW0522466 | 539.0 | 2.00 | 3.00 | 3 | 1 | 2 | 121.3 | 1 | 40 | 2 | 0 | 0 |
| 234 | sambuc | Lauterbach | 1901 | 1901 | WAG | WAG.1506268 | 492.1 | 1.00 | 2.00 | 2 | 1 | 7 | 113.1 | 1 | 35 | 3 | 1 | 0 |
| 235 | tilpla | Charbonnet-Salle | 1890 | 1890 | P | P04642486 | . | 3.50 | 4.00 | 2 | 0 | 9 | . | 1 | 30 | 3 | 0 | 0 |
| 236 | sambuc | de Bruyn | 1939 | 1939 | L | L.2973520 | 587.5 | 3.50 | 4.50 | 1 | . | 26 | 91.1 | 1 | 60 | 3 | 1 | 1 |
| 237 | corylu | Vaillant | b.1722 | 1722 | P | P06810674 | . | 5.00 | 5.00 | 3 | 1 | 6 | . | 1 | 40 | 3 | 0 | 0 |
| 238 | corylu | Woronow | 1905 | 1905 | TBI | TBI1005852 | 520.8 | 1.50 | 4.00 | 2 | 0 | 11 | 88.9 | 1 | 30 | 3 | 3 | 3 |
| 239 | querob | Zimmeter | 1888 | 1888 | MW | MW0771215 | 470.0 | 1.50 | 3.50 | 3 | 1 | 36 | 136.7 | 0 | 35 | 3 | 2 | 1 |
| 240 | alnglu | Wieringa | 2007 | 2007 | WAG | WAG.0220055 | 485.5 | 3.50 | 2.50 | 1 | . | 63 | 51.3 | 1 | 50 | 3 | 1 | 1 |
| 241 | tilpla | Valckenier Suringar | 1906 | 1906 | WAG | WAG.1857344 | 483.4 | 2.00 | 5.50 | 1 | . | 11 | 81.1 | 1 | 40 | 3 | 0 | 1 |
| 242 | poptre | Reutt | 1946 | 1946 | MW | MW0300869 | 500.7 | 4.50 | 2.00 | 1 | . | 41 | 35.6 | 0 | 40 | 3 | 1 | 0 |
| 243 | sambuc | Ososki & Saborio | 2000 | 2000 | NY | 1688394 | 510.8 | 3.00 | 4.00 | 1 | . | 2 | 151.7 | 1 | 25 | 3 | 1 | 0 |
| 244 | pyrus_ | Hess et al. | 2001 | 2001 | NY | 02854270 | 509.9 | 4.50 | 3.00 | 1 | . | 25 | 99.2 | 1 | 40 | 3 | 2 | 0 |
| 245 | betula | Pop | 1937 | 1937 | L | L.1558878 | 583.6 | 2.00 | 3.00 | 1 | . | 28 | 63.1 | 0 | 25 | 3 | 1 | 1 |
| 246 | poptre | Vonk | 1976 | 1976 | AMD | AMD.107815 | 536.8 | 2.50 | 3.00 | 2 | 1 | 39 | 48.5 | 0 | 30 | 3 | 1 | 0 |
| 247 | tilpla | Gyhr | 1920 | 1920 | BASBG | BASBG-00005188 | 490.3 | 3.50 | 4.00 | 1 | . | 6 | 88.8 | 1 | 25 | 2 | 0 | 1 |
| 248 | acetat | Nitka & Vytouš | 1958 | 1958 | P | P94765394 | . | 5.00 | 2.00 | 1 | . | 27 | . | 1 | 55 | 3 | 1 | 1 |
| 249 | acetat | Ekim | 1971 | 1971 | E | E00436961 | 493.1 | 3.50 | 3.00 | 1 | . | 31 | 75.8 | 1 | 50 | 3 | 2 | 3 |
| 250 | betula | Ehrhart; Don | b.1814 | 1814 | LINN | 1453.13 | 457.3 | 5.00 | 4.50 | 4 | 1 | 38 | 55.6 | 1 | 50 | 2 | 1 | 0 |
| 251 | acetat | Gmelin (?) | b.1755 | 1755 | LINN | 1225.4 | 386.0 | 2.70 | 4.30 | 2 | 0 | 10 | 86.7 | 1 | 40 | 3 | 0 | 1 |
| 252 | tilpla | Sherard | b.1728 | 1728 | OXF | . | 336.0 | 4.00 | 4.00 | 1 | . | 4 | 65.4 | 1 | 15 | 3 | 1 | 0 |
| 253 | alnglu | Biolog. stud. Wageningen | 1993 | 1993 | WAG | WAG.1868589 | 481.7 | 3.50 | 3.50 | 1 | . | 65 | 57.4 | 1 | 60 | 3 | 1 | 1 |
| 254 | acepla | Longbottom | 2008 | 2008 | NY | 02487611 | 506.7 | 5.00 | 4.50 | 1 | . | 4 | 102.8 | 1 | 20 | 3 | 1 | 0 |
| 255 | poptre | Żmujdzinowicz | 1876 | 1876 | MW | MW0300870 | 410.8 | 3.00 | 5.00 | 1 | . | 25 | 41.5 | 0 | 50 | 3 | 0 | 0 |
| 256 | acepla | Keough | 1980 | 1980 | WAG | WAG.1405222 | 485.9 | 3.50 | 4.00 | 1 | . | 5 | 177.6 | 1 | 50 | 3 | 1 | 1 |
| 257 | alnglu | Rose | b.1792 | 1792 | LINN | 1453.3 | 447.2 | 5.33 | 5.33 | 2 | 1 | 17 | 49.9 | 1 | 45 | 2 | 1 | 1 |
| 258 | corylu | Hepli | 1958 | 1958 | U | U.1155264 | 598.9 | 3.00 | 2.00 | 1 | . | 18 | 64.2 | 1 | 20 | 3 | 1 | 1 |
| 259 | tilpla | Linnaeus | b.1753 | 1753 | LINN | 679.1 | 370.4 | 4.50 | 1.50 | 1 | . | 14 | 80.0 | 1 | 50 | 3 | 0 | 1 |
| 260 | acetat | Poiret | b.1825 | 1825 | P | P06602538 | . | 4.50 | 5.00 | 1 | . | 6 | . | 1 | 30 | 2 | 0 | 1 |
| 261 | sambuc | Candolle | b.1822 | 1822 | P | P03707259 | . | 3.00 | 4.50 | 1 | . | 10 | . | 1 | 25 | 2 | 0 | 0 |
| 262 | sambuc | Krylov et al. | 2008 | 2008 | MW | MW0522465 | 515.0 | 1.00 | 2.00 | 1 | . | 11 | 150.8 | 0 | 50 | 3 | 1 | 0 |
| 263 | querob | . | 1802 | 1802 | MW | MW0771206 | 450.8 | 3.00 | 5.00 | 1 | . | 7 | 123.2 | 0 | 30 | 3 | 1 | 0 |
| 264 | poptre | Jovet | 1942 | 1942 | P | P02599453 | . | 3.00 | 2.50 | 3 | 1 | 107 | . | 1 | 75 | 3 | 1 | 0 |
| 265 | querob | Kuvaev & Jäger | 1978 | 1978 | MW | MW0771207 | 493.7 | 3.00 | 1.50 | 2 | 0 | 29 | 124.9 | 0 | 40 | 3 | 1 | 0 |
| 266 | poptre | . | c.1550 | 1550 | L | . | . | 1.67 | 4.33 | 1 | . | 28 | . | 0 | 25 | 3 | 1 | 0 |
| 267 | tilpla | Clute | 1898 | 1898 | NY | 02557505 | 516.1 | 1.00 | 2.00 | 2 | 0 | 9 | 72.7 | 0 | 25 | 2 | 0 | 0 |
| 268 | querob | . | b.1760 | 1760 | BM | BM000647424 | 379.9 | 3.00 | 2.67 | 1 | . | 34 | 124.5 | 0 | 40 | 3 | 1 | 0 |
| 270 | corylu | Sherard | b.1728 | 1728 | OXF | . | 440.2 | 4.50 | 3.00 | 2 | 1 | 7 | 68.1 | 1 | 25 | 3 | 0 | 0 |
| 271 | betula | Kuvaev | 1981 | 1981 | MW | MW0313976 | 518.0 | 1.50 | 1.50 | 1 | . | 38 | 46.8 | 0 | 30 | 3 | 1 | 0 |
| 272 | tilpla | Till | 2011 | 2011 | WU | WU0060876 | 524.4 | 1.50 | 6.00 | 1 | . | 14 | 102.6 | 0 | 40 | 3 | 1 | 0 |
| 273 | acetat | Maire | 1830 | 1830 | P | P05300054 | . | 4.00 | 3.00 | 4 | 0 | 40 | . | 1 | 50 | 3 | 1 | 3 |
| 274 | pyrus_ | Braun-Blanquet | 1932 | 1932 | L | L.1904033 | 582.7 | 3.00 | 3.50 | 1 | . | 33 | 34.7 | 0 | 20 | 3 | 1 | 0 |
| 275 | sambuc | Schiffner | 1911 | 1911 | L | L.2973517 | 538.3 | 6.00 | 2.50 | 4 | 1 | 6 | 134.5 | 1 | 60 | 3 | 1 | 1 |
| 276 | sambuc | Feuilleaubois | 1894 | 1894 | L | L.2973518 | 463.6 | 3.00 | 4.50 | 1 | . | 2 | 105.0 | 1 | 30 | 2 | 1 | 0 |
| 277 | sambuc | . | 1839 | 1839 | P | P04334262 | . | 3.50 | 2.50 | 1 | . | 6 | . | 1 | 40 | 3 | 1 | 0 |
| 278 | acetat | Tauscher | 1872 | 1872 | AMD | AMD.107250 | 479.1 | 1.50 | 3.50 | 2 | 1 | 34 | 81.6 | 1 | 40 | 3 | 1 | 1 |
| 279 | querob | Goldbach | 1817 | 1817 | MW | MW0315339 | 436.4 | 3.00 | 3.00 | 1 | . | 9 | 128.8 | 0 | 25 | 3 | 1 | 0 |
| 280 | alnglu | Nyárády & Bujorean | 1923 | 1923 | L | L.1560817 | 588.6 | 5.50 | 3.50 | 5 | 1 | 10 | 67.2 | 1 | 25 | 3 | 0 | 0 |
| 281 | sambuc | Dhetchuvi | 1991 | 1991 | WAG | WAG.1506263 | 495.5 | 1.00 | 1.50 | 1 | . | 14 | 136.7 | 1 | 50 | 3 | 1 | 1 |
| 282 | betula | Tschistiakoff | 1868 | 1868 | MW | MW0313944 | 521.6 | 4.00 | 3.50 | 1 | . | 43 | 35.2 | 0 | 20 | 3 | 1 | 0 |
| 283 | querob | Clason | 1956 | 1956 | L | L.1574494 | 510.7 | 2.00 | 3.50 | 3 | 1 | 6 | 97.1 | 1 | 20 | 1 | 0 | 1 |
| 284 | pyrus_ | Rock | 1925 | 1925 | P | P03132048 | . | 3.50 | 3.00 | 1 | . | 26 | . | 0 | 35 | 3 | 1 | 0 |
| 285 | sambuc | Rehder | 1920 | 1920 | K | K000898102 | 390.0 | 3.00 | 2.00 | 1 | . | 17 | 106.8 | 1 | 50 | 3 | 1 | 1 |
| 286 | betula | Clifford | b.1760 | 1760 | BM | BM000647388 | 521.1 | 4.67 | 4.00 | 1 | . | 54 | 51.4 | 0 | 30 | 3 | 1 | 0 |
| 287 | alnglu | Heimans | 1956 | 1956 | AMD | AMD.40755 | 538.8 | 2.00 | 1.50 | 3 | 2 | 22 | 52.0 | 1 | 45 | 3 | 3 | 3 |
| 288 | acetat | Baldacci | 1897 | 1897 | P | P05300059 | . | 3.50 | 3.00 | 3 | 0 | 26 | . | 1 | 40 | 3 | 1 | 1 |
| 289 | acepla | Linnaeus | b.1753 | 1753 | LINN | 1225.11 | 380.7 | 1.50 | 3.75 | 2 | 1 | 1 | 74.7 | 1 | 10 | 0 | 0 | 0 |
| 290 | sambuc | Segret | 1893 | 1893 | P | P00699345 | . | 6.00 | 3.50 | 3 | 1 | 6 | . | 1 | 50 | 3 | 1 | 1 |
| 291 | acepla | Nee | 1993 | 1993 | U | U.1579582 | 599.2 | 4.50 | 5.50 | 2 | 0 | 12 | 96.4 | 1 | 60 | 3 | 1 | 1 |
| 292 | sambuc | Buiten | 2013 | 2013 | L | L2072780 | 586.1 | 3.00 | 1.00 | 4 | 2 | 19 | 130.6 | 1 | 40 | 3 | 1 | 1 |
| 293 | poptre | Syutkina | 1988 | 1988 | MW | MW0300861 | 508.6 | 3.50 | 4.50 | 1 | . | 10 | 59.6 | 0 | 25 | 3 | 0 | 0 |
| 294 | querob | Welch | 1963 | 1963 | NY | 01465814 | 504.0 | 3.00 | 4.00 | 2 | 0 | 28 | 100.0 | 0 | 40 | 3 | 1 | 0 |
| 295 | poptre | Breteler | 1999 | 1999 | WAG | WAG.1210896 | 489.0 | 4.00 | 3.00 | 4 | 0 | 38 | 43.4 | 1 | 35 | 3 | 1 | 0 |
| 296 | acetat | Hohenacker | b.1841 | 1841 | E | E00436954 | . | 2.00 | 1.50 | 8 | 1 | 16 | . | 0 | 40 | 3 | 1 | 2 |
| 297 | tilpla | Audibert | 1823 | 1823 | K | K000687783 | 451.9 | 1.80 | 2.60 | 1 | . | 8 | 89.8 | 0 | 80 | 3 | 0 | 0 |
| 298 | corylu | Shuttleworth | 1835 | 1835 | BM | BM001191571 | 516.8 | 1.00 | 2.00 | 2 | 1 | 32 | 78.0 | 1 | 50 | 3 | 2 | 1 |
| 299 | betula | Ehrhart | 1785 | 1785 | MW | MW0591849 | 535.8 | 2.50 | 4.50 | 1 | . | 8 | 66.3 | 0 | 20 | 0 | 0 | 0 |
| 300 | tilpla | Vaillant | b.1722 | 1722 | P | P06700373 | . | 2.00 | 1.50 | 5 | 1 | 5 | . | 1 | 75 | 3 | 0 | 0 |
| 301 | poptre | von Mohl | 1811 | 1811 | MW | MW0770268 | 520.8 | 3.50 | 2.25 | 2 | 1 | 15 | 29.4 | 1 | 20 | 3 | 1 | 0 |
| 302 | sambuc | Coste & de Chamberet | 1915 | 1915 | P | P00724637 | . | 4.50 | 1.50 | 2 | 1 | 5 | . | 1 | 70 | 3 | 1 | 0 |
| 303 | sambuc | Laurence | 1948 | 1948 | P | P00321232 | . | 4.50 | 2.50 | 1 | . | 15 | . | 1 | 40 | 3 | 1 | 0 |
| 304 | sambuc | Szovits | 1829 | 1829 | P | P03707750 | . | 1.00 | 2.00 | 11 | 1 | 8 | . | 1 | 50 | 3 | 0 | 0 |
| 305 | corylu | Tschonoski | 1864 | 1864 | K | K000859897 | 437.5 | 1.00 | 5.50 | 2 | 1 | 5 | 73.6 | 0 | 15 | 1 | 0 | 2 |
| 306 | acepla | Flerow | 1893 | 1893 | MW | MW0691848 | 540.8 | 2.00 | 3.50 | 1 | . | 8 | 86.6 | 0 | 25 | 3 | 2 | 0 |
| 307 | querob | Duffour | 1895 | 1895 | BM | BM000613041 | 506.9 | 4.00 | 1.50 | 3 | 1 | 21 | 67.8 | 1 | 45 | 3 | 1 | 1 |
| 308 | tilpla | . | 1862 | 1862 | P | P04639439 | . | 4.00 | 5.50 | 1 | . | 4 | . | 1 | 20 | 2 | 0 | 0 |
| 309 | sambuc | Vaillant | b.1722 | 1722 | P | P03707755 | . | 4.00 | 4.25 | 1 | . | 6 | . | 1 | 60 | 3 | 0 | 0 |
| 310 | acepla | Bagley | 1967 | 1967 | AMD | AMD.107172 | . | 3.50 | 3.00 | 2 | 1 | 14 | . | 1 | 35 | 3 | 1 | 1 |
| 311 | betula | Kitaibel (?) | b.1817 | 1817 | GOET | GOET00589 | 535.0 | 2.00 | 6.00 | 2 | 2 | 50 | 35.6 | 1 | 20 | 3 | 1 | 1 |
| 312 | querob | Morse | 2010 | 2010 | NY | 02609488 | 517.4 | 5.50 | 3.50 | 2 | 0 | 37 | 109.8 | 1 | 55 | 3 | 1 | 0 |
| 313 | betula | Boom | 1954 | 1954 | L | L.1558803 | 583.3 | 4.00 | 3.50 | 3 | 0 | 60 | 51.4 | 1 | 50 | 3 | 1 | 1 |
| 314 | poptre | . | 1855 | 1855 | L | L.1544655 | 588.9 | 3.50 | 2.00 | 5 | 1 | 30 | 46.1 | 1 | 40 | 3 | 1 | 1 |
| 315 | betula | Trautvetter | 1844 | 1844 | LE | LE01051995 | 497.0 | 3.00 | 5.00 | 1 | . | 28 | 57.5 | 1 | 30 | 3 | 1 | 0 |
| 316 | acepla | Moldenke | 1935 | 1935 | NY | 02487527 | 507.5 | 5.50 | 1.50 | 1 | . | 16 | 117.6 | 1 | 75 | 3 | 1 | 2 |
| 317 | alnglu | Hohenacker | 1836 | 1836 | L | L.1560799 | 506.6 | 4.50 | 1.00 | 4 | 1 | 15 | 73.1 | 1 | 40 | 3 | 1 | 1 |
| 318 | acepla | Drushel | 1914 | 1914 | P | P05212312 | . | 5.00 | 1.00 | 2 | 2 | 12 | . | 1 | 50 | 3 | 1 | 1 |
| 319 | alnglu | Ten Broek | 1975 | 1975 | L | L.3173871 | 583.1 | 2.50 | 1.50 | 2 | 0 | 31 | 78.6 | 0 | 60 | 3 | 1 | 1 |
| 320 | pyrus_ | Ozanon & Gillot | 1882 | 1882 | L | L.1904028 | 585.8 | 3.00 | 4.50 | 2 | 1 | 16 | 36.1 | 1 | 10 | 3 | 3 | 2 |
| 321 | acetat | Attila | 1964 | 1964 | E | E00436966 | 500.2 | 3.00 | 3.00 | 1 | . | 17 | 74.4 | 1 | 25 | 3 | 1 | 1 |
| 322 | corylu | Sherard | b.1728 | 1728 | OXF | . | 433.3 | 5.50 | 5.75 | 1 | . | 9 | 81.7 | 1 | 40 | 3 | 1 | 0 |
| 323 | poptre | Ivanitzky | 1880 | 1880 | MW | MW0300868 | 379.6 | 1.50 | 2.00 | 1 | . | 29 | 36.7 | 0 | 35 | 3 | 1 | 0 |
| 324 | acepla | Schatz & Krause | 1892 | 1892 | MW | MW0852971 | 532.6 | 2.00 | 3.00 | 1 | . | 8 | 111.1 | 0 | 60 | 3 | 2 | 0 |
| 325 | alnglu | Delacour | 1878 | 1878 | L | L.1560823 | 585.3 | 2.50 | 3.50 | 9 | 1 | 22 | 61.9 | 1 | 40 | 2 | 2 | 1 |
| 326 | alnglu | Pourret | b.1818 | 1818 | P | P00536912 | . | 2.33 | 1.00 | 4 | 1 | 9 | . | 1 | 30 | 2 | 0 | 0 |
| 327 | corylu | Maack | 1855 | 1855 | P | P06810012 | . | 1.00 | 1.00 | 6 | 0 | 22 | . | 0 | 30 | 2 | 2 | 0 |
| 328 | poptre | Scheppig | 1852 | 1852 | AMD | AMD.107818 | 537.5 | 1.50 | 5.00 | 1 | . | 17 | 42.3 | 0 | 25 | 2 | 0 | 0 |
| 329 | alnglu | Blom | 1948 | 1948 | L | L.1560821 | 583.1 | 2.50 | 5.00 | 1 | . | 16 | 74.7 | 0 | 45 | 3 | 0 | 0 |
| 330 | acepla | Cintract | 1849 | 1849 | P | P00047706 | . | 3.00 | 4.50 | 1 | . | 4 | . | 1 | 40 | 0 | 2 | 1 |
| 331 | acepla | Poiret | b.1825 | 1825 | P | P05302716 | . | 3.50 | 4.00 | 4 | 1 | 4 | . | 1 | 30 | 1 | 1 | 1 |
| 332 | betula | . | 1765–1766 | 1766 | E | E00854347,  E00854348 | 496.0 | 2.50 | 2.50 | 2 | 0 | 63 | 30.6 | 1 | 30 | 3 | 1 | 1 |
| 333 | querob | Hatcher | 1875 | 1875 | NY | 01465812 | 493.7 | 4.00 | 2.00 | 3 | 0 | 14 | 74.8 | 1 | 50 | 3 | 1 | 0 |
| 334 | sambuc | de Wilde & Dorgelo | 1961 | 1961 | AMD | AMD.24654 | 539.0 | 1.00 | 1.00 | 1 | . | 12 | 168.5 | 1 | 50 | 3 | 3 | 1 |
| 335 | betula | Klinge | 1846 | 1846 | LE | LE01051998 | 512.1 | 2.00 | 1.50 | 1 | . | 13 | 92.3 | 0 | 25 | 3 | 1 | 1 |
| 336 | poptre | Clifford (?) | b.1760 | 1760 | WAG | . | . | 3.00 | 2.67 | 1 | . | 29 | . | 0 | 30 | 3 | 1 | 1 |
| 337 | poptre | de Koster et al. | 1951 | 1951 | AMD | AMD.107827 | 540.8 | 1.50 | 5.50 | 1 | . | 22 | 45.5 | 0 | 25 | 3 | 1 | 1 |
| 338 | acepla | Bersan | 1987 | 1987 | WAG | WAG.1405195 | 487.6 | 4.00 | 3.50 | 2 | 0 | 20 | 85.6 | 0 | 50 | 3 | 1 | 0 |
| 339 | sambuc | Sag | 1982 | 1982 | P | P00041024 | . | 6.00 | 1.50 | 2 | 1 | 2 | . | 1 | 40 | 3 | 0 | 1 |
| 340 | corylu | Ignatov & Petelin | 1977 | 1977 | MW | MW0049857 | 521.8 | 3.50 | 5.50 | 1 | . | 23 | 86.7 | 0 | 50 | 3 | 1 | 0 |
| 341 | acetat | Lönnbohm | 1902 | 1902 | AMD | AMD.107239 | 549.0 | 3.00 | 3.00 | 4 | 0 | 14 | 100.0 | 1 | 40 | 2 | 1 | 1 |
| 342 | corylu | Alboff | 1893 | 1893 | LE | LE01024008 | 494.6 | 3.00 | 3.50 | 2 | 2 | 19 | 84.1 | 0 | 45 | 3 | 2 | 2 |
| 343 | betula | Cherednichenko et al. | 2014 | 2014 | MW | MW0313325 | 496.7 | 3.00 | 3.50 | 1 | . | 25 | 84.8 | 0 | 40 | 3 | 1 | 0 |
| 344 | querob | Groen | 2005 | 2005 | WAG | WAG.1318408 | 525.4 | 2.50 | 2.00 | 2 | 0 | 8 | 106.2 | 0 | 30 | 1 | 1 | 0 |
| 345 | alnglu | Curtis | 1903 | 1903 | NY | 02392882 | . | 3.50 | 4.00 | 1 | . | 12 | . | 1 | 30 | 2 | 1 | 1 |
| 346 | acepla | Atha | 2014 | 2014 | NY | 02456812 | 505.1 | 2.00 | 3.50 | 1 | . | 8 | 149.9 | 0 | 70 | 3 | 1 | 1 |
| 347 | querob | Smith | 1813 | 1813 | LINN | 1478.20 | 488.7 | 4.33 | 3.33 | 2 | 0 | 20 | 111.4 | 1 | 70 | 3 | 2 | 2 |
| 348 | poptre | Fournie | 1922 | 1922 | P | P04026701 | 519.3 | 5.00 | 5.00 | 1 | . | 39 | 51.2 | 0 | 50 | 3 | 0 | 0 |
| 349 | acetat | Ehrhart | b.1795 | 1795 | LINN | 1592.4 | . | 4.33 | 2.67 | 2 | 0 | 10 | . | 1 | 50 | 3 | 1 | 2 |
| 350 | acepla | Poiret | b.1825 | 1825 | P | P05302766 | . | 4.00 | 4.50 | 2 | 1 | 1 | . | 1 | 20 | 0 | 0 | 1 |
| 351 | corylu | Vekhov | 1941 | 1941 | MW | MW0049867 | 522.9 | 4.50 | 2.50 | 1 | . | 18 | 75.1 | 1 | 40 | 3 | 2 | 1 |
| 352 | pyrus_ | Poiret | b.1825 | 1825 | P | P03206979 | . | 2.50 | 5.00 | 1 | . | 7 | . | 0 | 10 | 2 | 0 | 1 |
| 353 | acepla | Nyman | 1858 | 1858 | P | P04765384 | . | 4.75 | 3.25 | 3 | 1 | 10 | . | 1 | 30 | 3 | 2 | 1 |
| 354 | tilpla | Ball | 1835 | 1835 | K | K000687767, K000687768 | 498.1 | 4.50 | 3.50 | 2 | 1 | 24 | 87.2 | 1 | 60 | 3 | 2 | 1 |
| 355 | alnglu | Sintenis | 1892 | 1892 | AMD | AMD.40758 | 539.5 | 2.50 | 2.00 | 9 | 2 | 17 | 60.3 | 1 | 30 | 2 | 1 | 1 |
| 356 | acepla | Karpuzoglu | 1963 | 1963 | E | E00436931 | 496.7 | 3.50 | 2.00 | 2 | 0 | 7 | 135.7 | 1 | 50 | 3 | 0 | 2 |
| 357 | tilpla | Ronniger | 1933 | 1933 | P | P00848159 | 523.9 | 3.50 | 4.00 | 1 | . | 14 | 90.5 | 1 | 40 | 3 | 1 | 0 |
| 358 | acetat | Linnaeus | b.1753 | 1753 | LINN | 1225.2 | 369.0 | 3.80 | 4.00 | 1 | . | 9 | 72.6 | 1 | 50 | 3 | 0 | 2 |
| 359 | pyrus_ | Fritzsche | 1891 | 1891 | L | L.1904036 | 581.9 | 5.00 | 3.50 | 2 | 1 | 19 | 60.8 | 1 | 15 | 3 | 1 | 0 |
| 360 | alnglu | Straehler | 1872 | 1872 | L | L.1560814 | 570.5 | 4.00 | 2.00 | 8 | 1 | 15 | 73.0 | 1 | 30 | 2 | 1 | 0 |
| 361 | acetat | Maksymovych | 1824–1826 | 1826 | MW | MW0434079 | 454.2 | 3.00 | 2.50 | 2 | 0 | 7 | 74.6 | 1 | 20 | 1 | 0 | 1 |
| 362 | sambuc | Billot | 1846 | 1846 | WAG | WAG.1506256 | 499.8 | 4.50 | 3.00 | 1 | . | 5 | 123.8 | 1 | 45 | 3 | 1 | 0 |
| 363 | pyrus_ | Komarov | 1896 | 1896 | P | P03132046 | . | 4.50 | 2.00 | 1 | . | 31 | . | 0 | 60 | 3 | 1 | 0 |
| 364 | pyrus_ | Hoek | 1980 | 1980 | L | L.3258746 | 585.6 | 2.00 | 6.00 | 1 | . | 28 | 31.4 | 1 | 15 | 1 | 1 | 1 |
| 365 | querob | Fontana & Crosetti | 1910 | 1910 | AMD | AMD.74077 | 498.7 | 3.00 | 1.00 | 4 | 0 | 41 | 103.3 | 1 | 60 | 3 | 1 | 1 |
| 366 | acepla | Boschnjak | b.1820 | 1820 | MW | MW0433859 | 493.8 | 5.50 | 4.50 | 1 | . | 7 | 76.4 | 1 | 25 | 3 | 1 | 2 |
| 367 | betula | Komarov | 1897 | 1897 | LE | LE01003110 | 516.8 | 4.50 | 4.00 | 3 | 0 | 36 | 51.6 | 1 | 30 | 3 | 1 | 1 |
| 368 | querob | d’Alverny | 1909 | 1909 | P | P06847388 | 530.3 | 5.50 | 6.00 | 2 | 1 | 24 | 108.0 | 1 | 40 | 3 | 1 | 0 |
| 369 | acetat | Fogg | 1969 | 1969 | NY | 02490454 | 515.7 | 3.00 | 3.50 | 1 | . | 8 | 106.9 | 1 | 40 | 2 | 0 | 0 |
| 370 | poptre | Wilson | 1907–1909 | 1909 | K | K000592068 | 489.2 | 5.50 | 5.00 | 3 | 1 | 11 | 47.7 | 1 | 40 | 2 | 0 | 0 |
| 371 | pyrus_ | Oldham | 1863 | 1863 | K | K000758074 | 552.1 | 4.00 | 1.00 | 3 | 1 | 27 | 53.7 | 1 | 75 | 3 | 1 | 1 |
| 372 | alnglu | . | 1822 | 1822 | P | P00536944 | . | 4.75 | 4.25 | 3 | 1 | 17 | . | 1 | 25 | 2 | 1 | 1 |
| 373 | tilpla | Ullepitsch | 1895 | 1895 | L | L.2361449 | 583.6 | 3.00 | 1.50 | 2 | 1 | 24 | 97.2 | 1 | 60 | 3 | 1 | 1 |
| 374 | acepla | Amirkhanov | 1975 | 1975 | MW | MW0691847 | 508.4 | 5.50 | 3.00 | 1 | . | 9 | 100.3 | 1 | 60 | 3 | 1 | 2 |
| 375 | poptre | Detlaf | 1914 | 1914 | MW | MW0300876 | 518.1 | 2.50 | 5.50 | 2 | 0 | 16 | 29.7 | 0 | 15 | 2 | 1 | 0 |
| 376 | betula | Storozheva | 1968 | 1968 | LE | LE01052000 | 513.2 | 2.00 | 3.50 | 1 | . | 23 | 63.4 | 0 | 30 | 3 | 1 | 0 |
| 377 | poptre | Goldbach | 1818 | 1818 | MW | MW0770260 | 525.5 | 1.00 | 3.00 | 1 | . | 1 | 171.9 | 0 | 20 | 0 | 0 | 0 |
| 378 | pyrus_ | Nelson | 1995 | 1995 | NY | 00042342 | 517.9 | 6.00 | 4.50 | 5 | 1 | 13 | 99.9 | 1 | 50 | 2 | 1 | 0 |
| 379 | sambuc | Mena | 1983 | 1983 | NY | 02443518 | 514.8 | 3.50 | 2.50 | 1 | . | 13 | 126.5 | 1 | 30 | 3 | 1 | 0 |
| 380 | corylu | Madalski | 1938 | 1938 | P | P06809908 | . | 5.50 | 3.00 | 4 | 1 | 13 | . | 1 | 70 | 2 | 0 | 0 |
| 381 | betula | Schultz | 1850 | 1850 | WAG | WAG.1513678 | 495.3 | 4.50 | 2.00 | 4 | 1 | 37 | 60.6 | 1 | 30 | 2 | 1 | 0 |
| 382 | tilpla | Wirtgen | 1857 | 1857 | L | L.2361648 | 566.8 | 4.00 | 5.50 | 1 | . | 9 | 91.1 | 1 | 40 | 3 | 1 | 0 |
| 383 | poptre | Stainton & Henderson | 1960 | 1960 | E | E00448561 | 502.7 | 4.00 | 3.50 | 1 | . | 48 | 44.4 | 0 | 50 | 3 | 1 | 0 |
| 384 | querob | Addor | 1957 | 1957 | NY | 01465805 | 509.4 | 5.50 | 4.00 | 4 | 2 | 10 | 106.3 | 1 | 25 | 3 | 0 | 0 |
| 385 | corylu | Boccone (?) | c.1671 | 1671 | P | P06810661 | . | 6.00 | 3.50 | 3 | 0 | 16 | . | 1 | 70 | 3 | 0 | 1 |
| 386 | betula | Grintescu | 1922 | 1922 | MW | MW0770899 | 531.2 | 1.00 | 3.00 | 2 | 0 | 29 | 66.0 | 0 | 30 | 3 | 1 | 0 |
| 387 | alnglu | Rytov | 1903 | 1903 | MW | MW0314374 | 510.3 | 1.00 | 3.00 | 1 | . | 24 | 69.1 | 0 | 40 | 3 | 1 | 1 |
| 388 | poptre | Straehler | 1872 | 1872 | L | L.1544637 | 588.1 | 6.00 | 3.50 | 3 | 1 | 6 | 60.3 | 1 | 20 | 2 | 0 | 0 |
| 389 | poptre | Aymonin | 1977 | 1977 | P | P04678165 | . | 3.50 | 1.00 | 1 | . | 21 | . | 0 | 50 | 3 | 2 | 0 |
| 390 | corylu | Hopkingson | 1930 | 1930 | P | P06841034 | . | 1.50 | 2.50 | 1 | . | 7 | . | 1 | 25 | 3 | 0 | 0 |
| 391 | betula | Ehrhart | b.1795 | 1795 | LINN | 1453.14 | . | 3.50 | 4.00 | 3 | 1 | 19 | . | 1 | 20 | 2 | 1 | 0 |
| 392 | alnglu | Drake | b.1856 | 1856 | P | P00538493 | 541.0 | 1.50 | 2.00 | 1 | . | 27 | 75.3 | 0 | 45 | 3 | 3 | 3 |
| 393 | betula | Linnaeus | b.1753 | 1753 | LINN | 1109.1 | . | 1.50 | 2.00 | 1 | . | 41 | . | 0 | 40 | 3 | 1 | 0 |
| 394 | acepla | Hill | 1976 | 1976 | NY | 02487677 | 513.5 | 5.00 | 4.50 | 1 | . | 5 | 117.4 | 1 | 40 | 3 | 0 | 0 |
| 395 | querob | Vaillant | 1700 | 1700 | P | P06857412 | . | 2.00 | 5.00 | 3 | 2 | 15 | . | 0 | 40 | 3 | 1 | 2 |
| 396 | sambuc | . | 1852 | 1852 | AMD | AMD.24651 | 537.0 | 5.00 | 5.00 | 1 | . | 3 | 100.5 | 1 | 20 | 3 | 1 | 0 |
| 397 | tilpla | Gillett | 1939 | 1939 | NY | 02557497 | 516.0 | 5.50 | 3.50 | 2 | 0 | 16 | 104.5 | 1 | 70 | 3 | 0 | 0 |
| 398 | querob | Syreisczikov | 1924 | 1924 | MHA | MHA0047091 | . | 3.00 | 3.00 | 1 | . | 39 | . | 0 | 45 | 3 | 0 | 0 |
| 399 | poptre | . | 1801 | 1801 | LINN | 1546.16 | 448.2 | 5.75 | 3.25 | 12 | 1 | 40 | 52.7 | 1 | 75 | 3 | 1 | 1 |
| 400 | pyrus_ | . | 1892 | 1892 | MW | MW0739168 | 535.3 | 3.00 | 1.50 | 2 | 2 | 23 | 94.3 | 1 | 60 | 3 | 2 | 1 |
| 401 | alnglu | Cherednichenko et al. | 2014 | 2014 | MW | MW314349 | 495.7 | 1.00 | 3.00 | 1 | . | 15 | 101.1 | 0 | 50 | 3 | 0 | 0 |
| 402 | pyrus_ | Baird | 1984 | 1984 | NY | 02854239 | 514.7 | 5.00 | 5.50 | 2 | 1 | 24 | 73.9 | 1 | 50 | 2 | 2 | 0 |
| 403 | corylu | Wang et al. | 1986 | 1986 | L | L.1559573 | 586.1 | 5.00 | 5.50 | 1 | . | 11 | 97.2 | 1 | 40 | 2 | 0 | 0 |
| 404 | querob | Pedersen et al. | 1971 | 1971 | MW | MW0771202 | 595.0 | 5.00 | 4.00 | 1 | . | 50 | 88.6 | 1 | 30 | 3 | 1 | 1 |
| 405 | querob | Goldbach | 1816 | 1816 | MW | MW0315185 | 510.3 | 2.67 | 4.33 | 1 | . | 10 | 149.2 | 0 | 45 | 3 | 1 | 0 |
| 406 | alnglu | von Mohl | 1799 | 1799 | MW | MW0770941 | 531.6 | 1.67 | 1.33 | 3 | 0 | 13 | 56.5 | 1 | 25 | 2 | 1 | 0 |
| 407 | acetat | de Jong & Banaszczak | 1996 | 1996 | WAG | WAG.1964304 | 476.9 | 2.00 | 1.00 | 1 | . | 26 | 91.1 | 0 | 60 | 3 | 1 | 0 |
| 408 | betula | Hibon | 1905 | 1905 | P | P00528829 | . | 2.00 | 5.50 | 2 | 0 | 94 | . | 0 | 50 | 3 | 3 | 0 |
| 409 | sambuc | Cope | 2009 | 2009 | K | K000914391 | 489.8 | 1.00 | 2.50 | 2 | 0 | 5 | 169.0 | 0 | 40 | 3 | 2 | 0 |
| 410 | tilpla | Clifford | b.1760 | 1760 | BM | BM000628731 | 525.7 | 3.00 | 4.50 | 2 | 1 | 8 | 81.4 | 1 | 35 | 3 | 1 | 2 |
| 411 | tilpla | . | c.1550 | 1550 | L | . | . | 1.00 | 1.50 | 2 | 0 | 18 | . | 1 | 40 | 3 | 1 | 1 |
| 412 | acetat | Andrzejowski | 1868 | 1868 | P | P05215967 | 419.9 | 4.00 | 4.50 | 1 | . | 37 | 90.9 | 1 | 45 | 3 | 2 | 1 |
| 413 | corylu | Taylor | 1978 | 1978 | WAG | WAG.1334361 | 487.9 | 4.00 | 5.50 | 5 | 1 | 6 | 121.4 | 1 | 40 | 1 | 0 | 1 |
| 414 | acepla | Curtis | 1902 | 1902 | NY | 02487552 | 480.1 | 4.50 | 5.50 | 2 | 1 | 12 | 102.8 | 1 | 40 | 2 | 1 | 1 |
| 415 | acepla | Kemularia-Nathadze et al. | 1958 | 1958 | E | E00436939 | 494.0 | 2.00 | 4.00 | 1 | . | 5 | 114.8 | 0 | 30 | 1 | 1 | 0 |
| 416 | tilpla | Sherard | b.1728 | 1728 | OXF | . | 431.7 | 1.67 | 4.33 | 1 | . | 7 | 89.9 | 1 | 40 | 1 | 0 | 0 |
| 417 | querob | Samuelsson | 1920 | 1920 | L | L.1574480 | 584.2 | 6.00 | 2.00 | 2 | 0 | 38 | 69.4 | 1 | 30 | 3 | 1 | 0 |
| 418 | betula | Loret | 1851 | 1851 | P | P00528821 | . | 5.00 | 2.50 | 1 | . | 26 | . | 1 | 20 | 3 | 1 | 1 |
| 419 | tilpla | van Setten | 1980 | 1980 | WAG | WAG.1857356 | 496.8 | 3.00 | 2.00 | 1 | . | 15 | 74.4 | 1 | 40 | 3 | 1 | 2 |
| 420 | acepla | Burch et al. | 1971 | 1971 | U | U.1579583 | 597.8 | 5.50 | 2.50 | 1 | . | 12 | 88.1 | 1 | 35 | 3 | 1 | 2 |
| 421 | pyrus_ | Vakhrameeva | 1990 | 1990 | MW | MW0097703 | 520.2 | 3.00 | 4.50 | 1 | . | 18 | 78.1 | 0 | 30 | 2 | 0 | 1 |
| 422 | pyrus_ | Kaden | 1942 | 1942 | MW | MW0384774 | 512.1 | 3.00 | 2.50 | 1 | . | 16 | 97.7 | 0 | 40 | 2 | 0 | 0 |
| 423 | sambuc | Schiede | 1829 | 1829 | NY | 00180326 | 477.1 | 5.50 | 3.50 | 1 | . | 2 | 158.4 | 1 | 50 | 3 | 2 | 2 |
| 424 | corylu | Blinkworth | 1830–1839 | 1839 | BM | BM001191574 | 526.3 | 3.50 | 1.00 | 6 | 2 | 6 | 144.0 | 1 | 50 | 1 | 1 | 2 |
| 425 | acetat | Nash | 1904 | 1904 | NY | 02490449 | 515.3 | 1.00 | 1.00 | 1 | . | 28 | 62.0 | 0 | 45 | 3 | 2 | 1 |
| 426 | sambuc | Nkunga | 1979 | 1979 | WAG | WAG.1506262 | 493.3 | 1.50 | 1.00 | 1 | . | 9 | 137.9 | 1 | 60 | 3 | 1 | 0 |
| 427 | corylu | Bürger | 1825–1835 | 1835 | M | M0153659 | 550.3 | 2.00 | 4.00 | 3 | 0 | 15 | 109.7 | 0 | 70 | 3 | 1 | 1 |
| 428 | tilpla | Ned. flora Excurzsie | 1952 | 1952 | L | L.3327090 | 579.4 | 6.00 | 3.00 | 2 | 0 | 32 | 76.1 | 1 | 40 | 3 | 1 | 1 |
| 429 | sambuc | Clifford | b.1753 | 1753 | BM | BM000558416 | 494.7 | 4.25 | 5.25 | 1 | . | 8 | 62.4 | 1 | 35 | 3 | 1 | 1 |
| 430 | corylu | Turczaninow | 1831 | 1831 | K | K000543885 | 485.3 | 4.00 | 3.00 | 2 | 1 | 30 | 65.3 | 1 | 35 | 3 | 1 | 2 |
| 431 | betula | Sherard | b.1728 | 1728 | OXF | . | 439.2 | 4.00 | 2.00 | 7 | 1 | 52 | 50.0 | 1 | 40 | 3 | 1 | 1 |
| 432 | pyrus_ | Zhudova & Pokrovskaya | 1944 | 1944 | MW | MW0097700 | 516.1 | 2.50 | 2.00 | 8 | 2 | 16 | 79.4 | 0 | 35 | 2 | 2 | 0 |
| 433 | poptre | Groult | 1996 | 1996 | P | P05464118 | . | 5.00 | 6.00 | 1 | . | 11 | . | 0 | 25 | 3 | 0 | 0 |
| 434 | querob | Wijnands | 1968 | 1968 | AMD | AMD.74076 | 529.7 | 1.50 | 3.00 | 2 | 1 | 47 | 109.3 | 1 | 35 | 3 | 1 | 1 |
| 435 | sambuc | Ehrhart | 1787–1793 | 1793 | WU | WU0077693 | 538.2 | 4.00 | 5.67 | 1 | . | 2 | 82.6 | 1 | 15 | 3 | 0 | 0 |
| 436 | betula | Gorbunova | 1959 | 1959 | LE | LE01053001 | 510.4 | 3.00 | 2.50 | 3 | 1 | 23 | 57.5 | 0 | 25 | 3 | 1 | 0 |
| 437 | poptre | Ball | 1877 | 1877 | E | E00448565 | 499.2 | 4.00 | 4.00 | 3 | 2 | 18 | 66.7 | 0 | 30 | 2 | 0 | 0 |
| 438 | tilpla | Bauhin | b.1624 | 1624 | BAS | . | 440.8 | 1.00 | 2.50 | 1 | . | 4 | 106.0 | 0 | 25 | 2 | 0 | 0 |
| 439 | poptre | Bos | 1964 | 1964 | AMD | AMD.107816 | 536.0 | 3.00 | 2.00 | 2 | 1 | 31 | 41.3 | 0 | 30 | 2 | 1 | 0 |
| 440 | acetat | Lehnert | 1849 | 1849 | MW | MW0434035 | . | 1.50 | 3.00 | 6 | 0 | 20 | . | 1 | 20 | 2 | 1 | 1 |
| 441 | pyrus_ | Maximowicz | 1854–1859 | 1859 | K | K000758073 | 508.9 | 3.00 | 3.00 | 2 | 1 | 29 | 68.3 | 1 | 50 | 3 | 1 | 1 |
| 442 | acetat | Buia et al. | 1963 | 1963 | MW | MW0782941 | 529.5 | 4.50 | 4.00 | 2 | 2 | 27 | 59.7 | 1 | 50 | 3 | 1 | 0 |
| 443 | tilpla | Callier | 1926 | 1926 | AMD | AMD.89928 | 534.6 | 5.50 | 3.00 | 3 | 1 | 22 | 71.8 | 1 | 60 | 3 | 1 | 1 |
| 444 | querob | Janka | 1867 | 1867 | BM | BM000613024 | 445.8 | 1.00 | 1.00 | 1 | . | 14 | 116.0 | 1 | 25 | 3 | 0 | 1 |
| 445 | pyrus_ | Sherard | b.1728 | 1728 | OXF | . | 433.8 | 5.86 | 4.86 | 2 | 0 | 10 | 60.5 | 1 | 30 | 3 | 0 | 0 |
| 446 | alnglu | Belder | 1981 | 1981 | WAG | WAG.1512584 | 483.5 | 4.50 | 4.50 | 2 | 1 | 23 | 91.5 | 1 | 50 | 3 | 1 | 3 |
| 447 | pyrus_ | Matthew | 1980 | 1980 | L | L.1904057 | 582.5 | 3.50 | 1.00 | 2 | 2 | 7 | 60.6 | 1 | 15 | 2 | 0 | 0 |
| 448 | betula | Rose | b.1792 | 1792 | LINN | 1453.11 | 443.7 | 3.50 | 3.00 | 3 | 1 | 20 | 65.3 | 1 | 20 | 3 | 2 | 2 |
| 449 | sambuc | Sargent | 1913 | 1913 | K | K000898103 | 497.1 | 2.50 | 3.50 | 3 | 2 | 5 | 151.5 | 1 | 60 | 3 | 1 | 2 |
| 450 | querob | Ekzertseva | 1977 | 1977 | MW | MW0315046 | 523.9 | 6.00 | 3.00 | 1 | . | 39 | 122.2 | 1 | 60 | 3 | 0 | 0 |
| 451 | poptre | van de Beek | 2013 | 2013 | L | L.2068012 | 578.3 | 2.50 | 2.00 | 1 | . | 51 | 40.0 | 0 | 40 | 3 | 1 | 0 |
| 452 | corylu | Bush | 1924 | 1924 | K | K000543893 | 488.5 | 6.00 | 1.50 | 2 | 1 | 22 | 98.1 | 1 | 70 | 3 | 1 | 1 |
| 453 | betula | Tikhomirov et al. | 1973 | 1973 | MW | MW0313344 | 525.0 | 6.00 | 5.00 | 2 | 0 | 33 | 53.4 | 1 | 30 | 2 | 0 | 0 |
| 454 | querob | Henning | b.1838 | 1838 | MW | MW0315086 | 485.7 | 1.50 | 4.00 | 1 | . | 22 | 107.0 | 0 | 30 | 3 | 1 | 0 |
| 455 | acetat | Kotschy | 1859 | 1859 | S | S11-22256 | 528.8 | 2.00 | 1.00 | 2 | 2 | 31 | 71.7 | 1 | 50 | 3 | 2 | 3 |
| 456 | poptre | Gillot | 1898 | 1898 | P | P05599618 | . | 3.50 | 5.00 | 1 | . | 39 | . | 0 | 40 | 3 | 1 | 0 |
| 457 | querob | Prilipko | 1927 | 1927 | L | L.1574454 | 521.4 | 3.00 | 5.50 | 3 | 1 | 21 | 120.8 | 1 | 60 | 3 | 0 | 0 |
| 458 | pyrus_ | Gentry | 1962 | 1962 | NY | 02854237 | 512.6 | 2.50 | 6.00 | 1 | . | 17 | 73.1 | 0 | 30 | 2 | 1 | 0 |
| 459 | acetat | Verhoek-Williams & Davis | 1970 | 1970 | NY | 02490469 | 514.2 | 5.00 | 3.50 | 1 | . | 27 | 75.1 | 1 | 35 | 3 | 0 | 1 |
| 460 | sambuc | Spencer & Dennis | 2007 | 2007 | BM | BM000954723 | 521.5 | 4.00 | 3.00 | 1 | . | 11 | 91.8 | 1 | 40 | 3 | 3 | 1 |
| 461 | corylu | Bunge | 1823 | 1823 | P | P06810657 | . | 1.60 | 3.60 | 4 | 1 | 13 | . | 1 | 50 | 3 | 1 | 2 |
| 462 | acetat | Wiegand & Cipperley | 1905 | 1905 | NY | 02490458 | 514.8 | 1.50 | 5.50 | 2 | 0 | 12 | 89.0 | 1 | 40 | 2 | 1 | 1 |
| 463 | alnglu | Yaichnikov | 1907 | 1907 | MW | MW0314347 | 526.5 | 1.50 | 5.00 | 1 | . | 8 | 59.2 | 0 | 20 | 2 | 1 | 1 |
| 464 | alnglu | Segal & Laak | 1965 | 1965 | AMD | AMD.40747 | 532.0 | 3.00 | 3.00 | 2 | 2 | 26 | 75.8 | 0 | 45 | 3 | 2 | 1 |
| 465 | poptre | Kukkonen | 1972 | 1972 | E | E00448566 | 488.6 | 3.00 | 2.00 | 1 | . | 54 | 43.2 | 0 | 40 | 3 | 1 | 1 |
| 466 | sambuc | Shepard | 1873 | 1873 | BM | BM001161533 | 535.8 | 2.50 | 4.00 | 1 | . | 5 | 137.3 | 1 | 30 | 3 | 2 | 0 |
| 467 | alnglu | Nee | 1993 | 1993 | NY | 02332750 | 504.7 | 5.00 | 2.50 | 3 | 0 | 22 | 91.5 | 1 | 60 | 3 | 0 | 1 |
| 468 | acepla | Jussieu | 1841 | 1841 | P | P00047723 | . | 3.00 | 3.50 | 1 | . | 8 | . | 1 | 60 | 3 | 2 | 1 |
| 469 | poptre | Pourret | 1818 | 1818 | P | P04668872 | . | 2.25 | 3.25 | 1 | . | 17 | . | 0 | 35 | 2 | 1 | 0 |
| 470 | betula | Lindquist | 1945 | 1945 | S | S-G-947 | 495.9 | 3.50 | 1.50 | 4 | 2 | 26 | 53.3 | 1 | 25 | 3 | 1 | 0 |
| 471 | sambuc | Arnolds | 1963 | 1963 | U | U.1056593 | 600.0 | 2.50 | 4.00 | 1 | . | 4 | 143.3 | 1 | 25 | 3 | 3 | 0 |
| 472 | corylu | Dudov & Kozhin | 2016 | 2016 | MW | MW0160431 | 505.4 | 5.00 | 1.50 | 2 | 1 | 28 | 89.2 | 1 | 75 | 3 | 1 | 0 |
| 473 | tilpla | Krylov | 2005 | 2005 | MW | MW0435840 | 497.2 | 2.00 | 2.50 | 1 | . | 11 | 117.9 | 0 | 70 | 3 | 1 | 1 |
| 474 | pyrus_ | Solander | b.1760 | 1760 | S | S09-28504 | 381.9 | 2.67 | 3.00 | 1 | . | 10 | 49.2 | 1 | 25 | 3 | 1 | 0 |
| 475 | acetat | Margittai | 1926 | 1926 | P | P05215972 | . | 2.50 | 2.00 | 2 | 0 | 24 | . | 1 | 40 | 3 | 1 | 1 |
| 476 | tilpla | Oertel | 1864 | 1864 | WAG | WAG.1856853 | 478.9 | 3.00 | 3.50 | 2 | 0 | 5 | 94.0 | 1 | 40 | 2 | 0 | 1 |
| 477 | pyrus_ | Scheppig | 1883 | 1883 | AMD | AMD.94945 | 538.3 | 5.00 | 1.50 | 2 | 1 | 39 | 39.0 | 1 | 30 | 3 | 1 | 1 |
| 478 | querob | Sherard | b.1728 | 1728 | OXF | . | 521.9 | 2.50 | 2.00 | 2 | 0 | 25 | 111.7 | 1 | 60 | 3 | 1 | 1 |
| 479 | alnglu | Heukels | 1980 | 1980 | L | L.1560798 | 571.6 | 4.50 | 2.50 | 2 | 2 | 15 | 73.5 | 1 | 25 | 3 | 1 | 0 |
| 480 | acepla | Vaillant | b.1722 | 1722 | P | P05302755 | . | 5.50 | 2.25 | 4 | 0 | 12 | . | 1 | 65 | 3 | 0 | 1 |
| 481 | acepla | Broekens | 1923 | 1923 | AMD | AMD.107169 | 541.2 | 5.50 | 3.00 | 1 | . | 13 | 103.6 | 1 | 40 | 3 | 2 | 1 |
| 482 | corylu | Karo | 1898 | 1898 | L | L.1559571 | 584.7 | 5.50 | 3.50 | 3 | 1 | 6 | 91.4 | 1 | 50 | 1 | 1 | 1 |
| 483 | poptre | Spach | 1832 | 1832 | P | P00981347 | 520.9 | 3.00 | 5.50 | 1 | . | 52 | 42.9 | 0 | 40 | 2 | 1 | 0 |
| 484 | tilpla | Candolle | 1807 | 1807 | GDC | G00209277 | 476.8 | 3.00 | 4.00 | 1 | . | 11 | 86.4 | 1 | 35 | 3 | 1 | 2 |
| 485 | alnglu | Sherard | b.1728 | 1728 | OXF | . | 430.1 | 4.60 | 3.80 | 2 | 1 | 17 | 61.1 | 1 | 45 | 3 | 0 | 1 |
| 486 | querob | Spencer & Dennis | 2007 | 2007 | BM | BM000954710 | 513.6 | 2.50 | 6.00 | 1 | . | 24 | 94.5 | 0 | 40 | 3 | 1 | 1 |
| 487 | betula | Tzvelev | 2001 | 2001 | LE | LE01051999 | 500.2 | 2.50 | 2.50 | 2 | 1 | 60 | 69.5 | 0 | 50 | 3 | 1 | 0 |
| 488 | acetat | Candolle | b.1824 | 1824 | GDC | G00211441 | 523.5 | 3.33 | 4.67 | 1 | . | 17 | 85.7 | 1 | 50 | 3 | 0 | 2 |
| 489 | querob | Boschnjak | 1820–1825 | 1825 | MW | MW0315338 | 534.9 | 2.67 | 2.67 | 1 | . | 11 | 90.7 | 0 | 50 | 3 | 2 | 1 |
| 490 | querob | Wirtgen | 1862 | 1862 | L | L.1574483 | 526.2 | 3.50 | 3.50 | 2 | 1 | 48 | 133.6 | 0 | 40 | 3 | 1 | 0 |
| 491 | betula | Puring | 1896 | 1896 | LE | LE01051997 | 502.6 | 3.00 | 5.00 | 2 | 0 | 49 | 43.2 | 0 | 30 | 3 | 1 | 0 |
| 492 | corylu | Vaillant | 1703 | 1703 | P | P06810673 | . | 6.00 | 2.50 | 2 | 1 | 3 | . | 1 | 25 | 0 | 0 | 0 |
| 493 | poptre | Linnaeus | b.1774 | 1774 | LINN | 1185.3 | 249.7 | 2.33 | 4.00 | 1 | . | 7 | 37.7 | 0 | 30 | 1 | 0 | 0 |
| 494 | acetat | Delendick | 1976 | 1976 | NY | 02490447 | 521.1 | 3.50 | 5.50 | 1 | . | 15 | 88.9 | 1 | 50 | 3 | 2 | 1 |
| 495 | betula | Jahn | 1910 | 1910 | AMD | AMD.40523 | 539.3 | 2.00 | 1.00 | 3 | 1 | 44 | 48.5 | 1 | 30 | 3 | 1 | 0 |
| 496 | acepla | Bauhin | b.1624 | 1624 | BAS | . | 460.4 | 1.33 | 3.00 | 1 | . | 4 | 73.6 | 0 | 25 | 1 | 0 | 1 |
| 498 | sambuc | Burbridge | 1960 | 1960 | P | P02409645 | . | 2.50 | 5.00 | 1 | . | 5 | . | 1 | 30 | 3 | 1 | 1 |
| 499 | querob | Bochkin & Majorov | 2012 | 2012 | MHA | MHA0047112 | 440.1 | 1.50 | 5.00 | 1 | . | 18 | 86.2 | 1 | 50 | 3 | 1 | 0 |
| 500 | betula | Segret | 1902 | 1902 | P | P00528855 | . | 5.50 | 5.50 | 1 | . | 55 | . | 1 | 60 | 3 | 1 | 0 |
| 501 | querob | Franco | 1951 | 1951 | BM | BM000613044 | 522.4 | 2.50 | 3.00 | 4 | 1 | 13 | 129.2 | 1 | 25 | 3 | 1 | 1 |
| 502 | pyrus_ | Baytop | 1961 | 1961 | E | E00408584 | 489.4 | 3.00 | 4.00 | 1 | . | 18 | 40.0 | 0 | 20 | 2 | 1 | 0 |
| 503 | betula | van der Burgh | 1997 | 1997 | U | U.1144113 | 596.1 | 1.00 | 5.00 | 1 | . | 107 | 39.7 | 0 | 40 | 3 | 1 | 1 |
| 504 | tilpla | Zahlbruckner | 1832 | 1832 | K | K000687769 | . | 2.50 | 4.00 | 1 | . | 3 | . | 1 | 30 | 2 | 0 | 1 |
| 505 | alnglu | Bouby | 1972 | 1972 | P | P00572468 | . | 5.00 | 5.00 | 3 | 1 | 16 | . | 1 | 75 | 3 | 1 | 0 |
| 506 | corylu | Borbás | 1895 | 1895 | P | P06809854 | . | 3.00 | 2.50 | 1 | . | 16 | . | 1 | 60 | 3 | 1 | 1 |
| 507 | acepla | Seregin | 2014 | 2014 | MW | MW0618521 | 514.6 | 1.50 | 3.50 | 2 | 2 | 8 | 100.3 | 0 | 50 | 2 | 2 | 1 |
| 508 | acepla | Ehrhart | b.1795 | 1795 | LINN | 1592.10 | 438.9 | 2.50 | 3.00 | 2 | 1 | 1 | 101.1 | 1 | 15 | 0 | 0 | 0 |
| 509 | acepla | Rodriguez | 1917 | 1917 | P | P00056500 | . | 3.00 | 6.00 | 1 | . | 4 | . | 0 | 20 | 2 | 1 | 0 |
| 510 | alnglu | van den Bosch | 1833 | 1833 | WAG | WAG.1512605 | 491.3 | 3.00 | 1.50 | 4 | 1 | 9 | 63.0 | 1 | 25 | 1 | 1 | 1 |
| 511 | querob | Bonpland | 1810 | 1810 | P | P06857435 | 526.0 | 1.00 | 1.75 | 1 | . | 42 | 132.6 | 0 | 30 | 3 | 1 | 0 |
| 512 | alnglu | Seyrat | 1914 | 1914 | P | P00536940 | . | 5.50 | 4.50 | 3 | 0 | 32 | . | 1 | 60 | 3 | 1 | 1 |
| 513 | corylu | Iwatsuki | 1965 | 1965 | L | L.1559580 | 581.7 | 1.50 | 2.00 | 1 | . | 23 | 98.1 | 1 | 70 | 3 | 1 | 2 |
| 514 | tilpla | Wagner | 1932 | 1932 | W | W1962-0008747 | 529.5 | 5.50 | 2.00 | 1 | . | 13 | 73.0 | 1 | 40 | 3 | 1 | 0 |
| 515 | alnglu | Scheppig | 1856 | 1856 | AMD | AMD.40752 | . | 1.50 | 2.50 | 2 | 0 | 10 | . | 1 | 25 | 2 | 1 | 0 |
| 516 | acepla | Candolle | b.1824 | 1824 | GDC | G00211694 | 528.3 | 4.00 | 2.50 | 5 | 1 | 6 | 138.3 | 1 | 40 | 2 | 1 | 1 |
| 517 | acetat | Sperling et al. | 1985 | 1985 | E | E00436957 | 474.4 | 4.50 | 6.00 | 1 | . | 13 | 66.1 | 1 | 30 | 3 | 1 | 1 |
| 518 | betula | Vysokikh et al. | 2000 | 2000 | LE | LE01051991 | 512.5 | 2.00 | 3.00 | 1 | . | 27 | 82.1 | 0 | 40 | 3 | 1 | 0 |
| 519 | pyrus_ | Koch | b.1849 | 1849 | L | L.1904051 | 585.6 | 4.50 | 5.00 | 1 | . | 31 | 42.3 | 1 | 20 | 3 | 1 | 0 |
| 520 | acepla | Szovits | 1827–1830 | 1830 | E | E00436943 | 486.4 | 1.67 | 4.67 | 1 | . | 5 | 116.8 | 0 | 50 | 2 | 0 | 1 |
| 521 | poptre | Davis | 1947 | 1947 | E | E00448559 | 498.2 | 5.00 | 3.00 | 1 | . | 34 | 53.5 | 0 | 60 | 3 | 1 | 0 |
